# Development of MAPT S305 mutation human iPSC lines exhibiting elevated 4R tau expression and functional alterations in neurons and astrocytes

**DOI:** 10.1016/j.celrep.2024.115013

**Published:** 2024-11-27

**Authors:** Kathryn R. Bowles, Chiara Pedicone, Derian A. Pugh, Laura-Maria Oja, Filipa H. Sousa, Lois K. Keavey, Brian Fulton-Howard, Sarah A. Weitzman, Yiyuan Liu, Jonathan L. Chen, Matthew D. Disney, Alison M. Goate

**Affiliations:** 1 Department of Genetics and Genomic Sciences, Icahn School of Medicine at Mount Sinai, New York, NY, USA; 2 Ronald M. Loeb Center for Alzheimer’s Disease, Icahn School of Medicine at Mount Sinai, New York, NY, USA; 3 UK Dementia Research Institute at The University of Edinburgh, Edinburgh Medical School, Edinburgh, UK; 4 Centre for Discovery Brain Sciences, School of Biomedical Sciences, College of Medicine and Veterinary Medicine, The University of Edinburgh, Edinburgh, UK; 5 Department of Chemistry, Scripps Research Institute, Jupiter, FL, USA; 6 Present address: UK Dementia Research Institute at The University of Edinburgh, Edinburgh Medical School, Edinburgh, UK; 7 Present address: University of Alabama at Birmingham, Birmingham, AL, USA; 8 Present address: BrainXell Therapeutics, San Diego, CA, USA; 9 Present address: Center for RNA Biology, University of Rochester Medical Center, Rochester, NY, USA; 10 Lead contact

## Abstract

Due to the importance of 4R tau (with four microtubule-binding-repeat domains) in the pathogenicity of primary tauopathies, it has been challenging to model these diseases in induced pluripotent stem cell (iPSC)-derived neurons, which express very low levels of 4R tau. To address this, we have developed a panel of isogenic iPSC lines carrying *MAPT* splice-site mutations, S305S, S305I, or S305N, derived from four different donors. All mutations significantly increase 4R tau expression in iPSC neurons and astrocytes. Functional analyses of S305 mutant neurons reveal shared disruption in synaptic signaling and maturity but divergent effects on mitochondrial bioenergetics. In iPSC astrocytes, S305 mutations promote internalization of exogenous tau that may be a precursor to glial pathology. These lines recapitulate previously characterized tauopathy-relevant phenotypes and highlight functional differences between the wild-type 4R and the mutant 4R proteins in both neurons and astrocytes. As such, these lines enable a more complete understanding of pathogenic mechanisms underlying 4R tauopathies across different cell types.

## In brief

Bowles et al. introduce an isogenic panel of human iPSC lines spanning three *MAPT* S305 splicing mutations that cause familial FTD that express high levels of 4R tau early in neuronal differentiation. Transcriptomic and functional characterization reveal synaptic and mitochondrial dysfunction in neurons and inflammation and tau internalization in astrocytes.

## Graphical Abstract

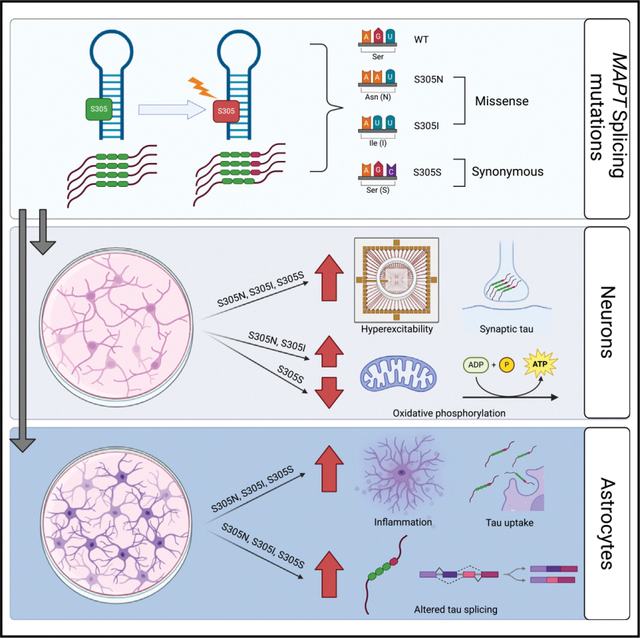

## INTRODUCTION

The *MAPT* gene, encoding the protein tau, is alternatively spliced at its N- and C-terminal ends, resulting in the expression of six different isoforms in the human brain. These can be categorized into two groups, dependent on the inclusion or exclusion of exon 10; inclusion results in four microtubule-binding-repeat domains (4R tau), whereas exclusion results in only three (3R tau). The 4R:3R tau ratio in healthy adult human brain is roughly 1:1,^1–3^ while expression in the developing fetal brain is almost exclusively 3R.^4^ Several primary tauopathies, including progressive supranuclear palsy (PSP), corticobasal degeneration (CBD), and some forms of frontotemporal lobar dementia-*MAPT* (FTLD-*MAPT*), are considered “4R tauopathies” due to the specific accumulation of this isoform in neuronal and astrocytic inclusions.^5^ The functional impact of aberrantly increased 4R tau expression and accumulation on cellular function is not fully understood, although these processes are likely to be key for determining mechanisms of disease pathogenesis and subsequent therapeutic intervention.

Human induced pluripotent stem cell (iPSC) models are valuable tools for the study of early cellular perturbations and mechanisms contributing to neurodegenerative disorders: for example, both 2D neuronal monocultures and 3D organoid models have recapitulated key phenotypes associated with tauopathies and reveal unique phenotypes of interest.^6–17^ However, these models, like other iPSC-derived neuronal models, most closely resemble fetal brain^18^ and therefore express almost exclusively 3R tau. Modeling the functional effect of aberrantly expressed 4R tau and pathogenic mechanisms that underlie 4R tauopathies has therefore been challenging in these systems.

To date, there have been numerous efforts to increase the expression of 4R tau in iPSC models, with mixed results.^19–23^ However, these models require extended periods in culture or complex culturing techniques and manipulations to achieve reasonable expression of 4R tau.^19–21^ While neurons derived from directly differentiated human fibroblasts more closely recapitulate signatures of aging and express 4R tau at a level equivalent to that of adult human brain,^22,23^ this model is limited in its capacity for expansion, isogenic correction, and accessibility to the wider scientific field.

To address this issue, we developed an isogenic set of iPSC lines with autosomal dominant S305 mutations, which cause FTLD-*MAPT*.^24–27^ S305 is the last codon of *MAPT* exon 10 and is therefore part of the RNA hairpin-loop structure that regulates exon 10 alternative splicing.^28^ Three mutations have been identified in this codon: two missense mutations (S305N and S305I) and one synonymous mutation (S305S) that all alter *MAPT* splicing, increasing exon 10 inclusion. However, while each clinical case has been described as a variant of frontotemporal dementia (FTD), each mutation is varied in its clinical and neuropathological presentation.^24–27^

Here, we present an isogenic panel of iPSCs carrying an S305N, S305I, or S305S mutation, derived from four independent donors. Each isogenic series includes the wild type (WT) and heterozygous and homozygous mutations. We demonstrate that these mutations significantly increase 4R tau expression in iPSC neurons and astrocytes from as early as 4 weeks of differentiation. Furthermore, we have characterized both shared and divergent cellular phenotypes associated with increased 4R tau between missense and synonymous splicing mutations, converging on synaptic function and mitochondrial metabolism in neurons and phagocytic function and inflammatory state in astrocytes. These lines are a valuable and necessary tool for progressing tauopathy research and are freely available to the scientific community.

## RESULTS

### Generation of an isogenic panel of *MAPT* S305 mutation iPSC lines

**Figure 1.**
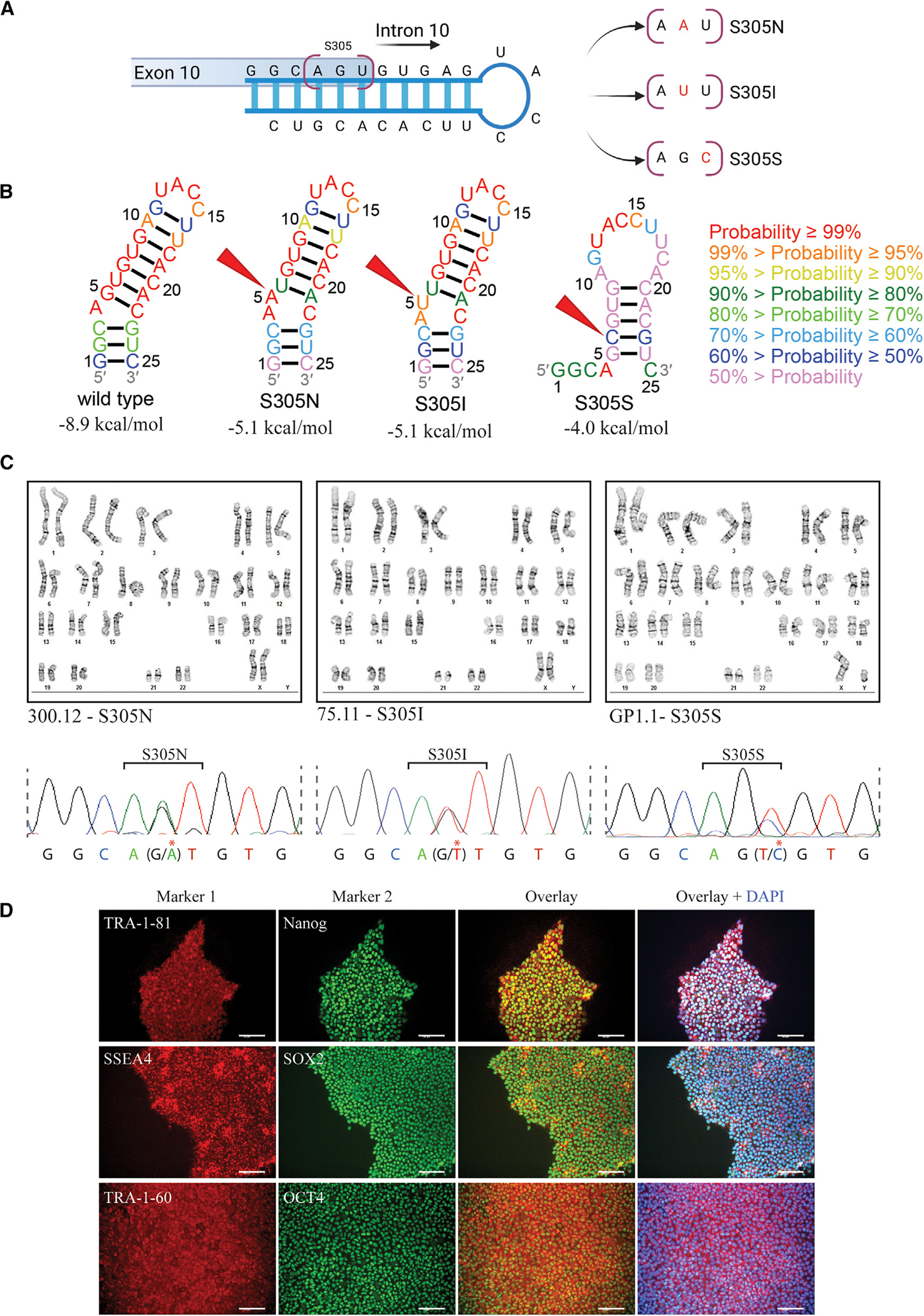
Generation of *MAPT* S305 mutation iPSC lines (A) Schematic of the *MAPT* exon 10-intron 10 RNA hairpin. (B) RNAstructure-predicted probability of base-paired and single-stranded nucleotides in WT and S305 mutation sequences. Modifications are indicated with a red arrow. Structure predictions were carried out using RNAstructure.^29^ (C) G-band karyotyping of reprogrammed parent clones selected for CRISPR editing from each *MAPT* mutation donor, paired with sequencing traces confirming heterozygote S305 mutation status. Red asterisks denote the mutation. (D) Representative example of pluripotency markers in reprogrammed iPSC clones. Scale bar, 100 μm.

iPSC-derived models of tauopathy are notoriously imperfect due to low expression of *MAPT* exon 10 and, consequently, of 4R tau. To address this, we generated iPSC lines with *MAPT* S305 splice-site mutations (Figures 1A and 1B) to increase exon 10 inclusion in these models. We identified peripheral blood mononuclear cells (PBMCs) from individuals heterozygous for *MAPT* S305I and S305N mutations as part of the ALLFTD study, as well as lymphoblastoid cell lines (LCLs) from an S305S mutation carrier at the Sydney Brain Bank (SBB, Sydney, Australia) (Table 1). PBMCs and LCLs were reprogrammed to iPSCs using Sendai-virus-mediated gene transfer of *OCT4*, *SOX2*, *KFL4*, and c-*MYC* at the Icahn School of Medicine at the Mount Sinai Stem Cell core facility. Following reprogramming, mutation status, karyotypic normality, and expression of pluripotency markers of one clone per donor were confirmed and selected for CRISPR-Cas9 genome editing (Figures 1C and 1D).

As donor and clonal variability are major sources of inconsistency in iPSC phenotypic characterization,^30^ we generated isogenic corrected WT controls from each donor line using CRISPR-Cas9 genome editing (Tables 1 and S1; Figure S1). We also edited each donor line to be homozygous for its original mutation (Table 1; Figure S1). To control for clonal variability and selective pressure, additional unedited clones per donor line were retained (Table 1; Figure S1). An additional WT iPSC line derived from a cognitively normal individual was selected for CRISPR-Cas9 induction of each S305 mutation onto the same control background (Tables 1 and S1; Figure S1). This resulted in a panel of 26 iPSC lines, derived from four donors, which are heterozygous or homozygous for *MAPT* S305S, S305I, or S305N splicing mutations (Table 1). Four clones were previously reported as a resource available from the Tau Consortium^31^ and are now included in the wider panel of S305 mutation lines reported here. All lines are freely available upon request from the National Centralized Repository for Alzheimer’s Disease (NCRAD; https://ncrad.iu.edu/) or the Tau Consortium via the Neural Stem Cell Institute (NSCI; http://neuralsci.org/tau).

### *MAPT* S305 mutation neurons express 4R tau from 4 weeks of differentiation

To date, iPSC neuron models of tauopathy have been unable to fully model the effect of increased 4R tau as observed in PSP and FTD brain, due to their similarity to fetal-age cells that express primarily the 3R isoform.^18–20^ *MAPT* S305 mutations were predicted to destabilize the exon 10-intron 10 RNA hairpin loop (Figure 1B), thus requiring less energy to open the loop. This promotes accessibility to cellular splicing machinery, thereby increasing exon 10 inclusion. As such, these mutations are neuropathologically associated with the accumulation of 4R tau.^24–27^ We therefore predicted these mutations would facilitate higher 4R tau expression in iPSC models.

**Figure 2.**
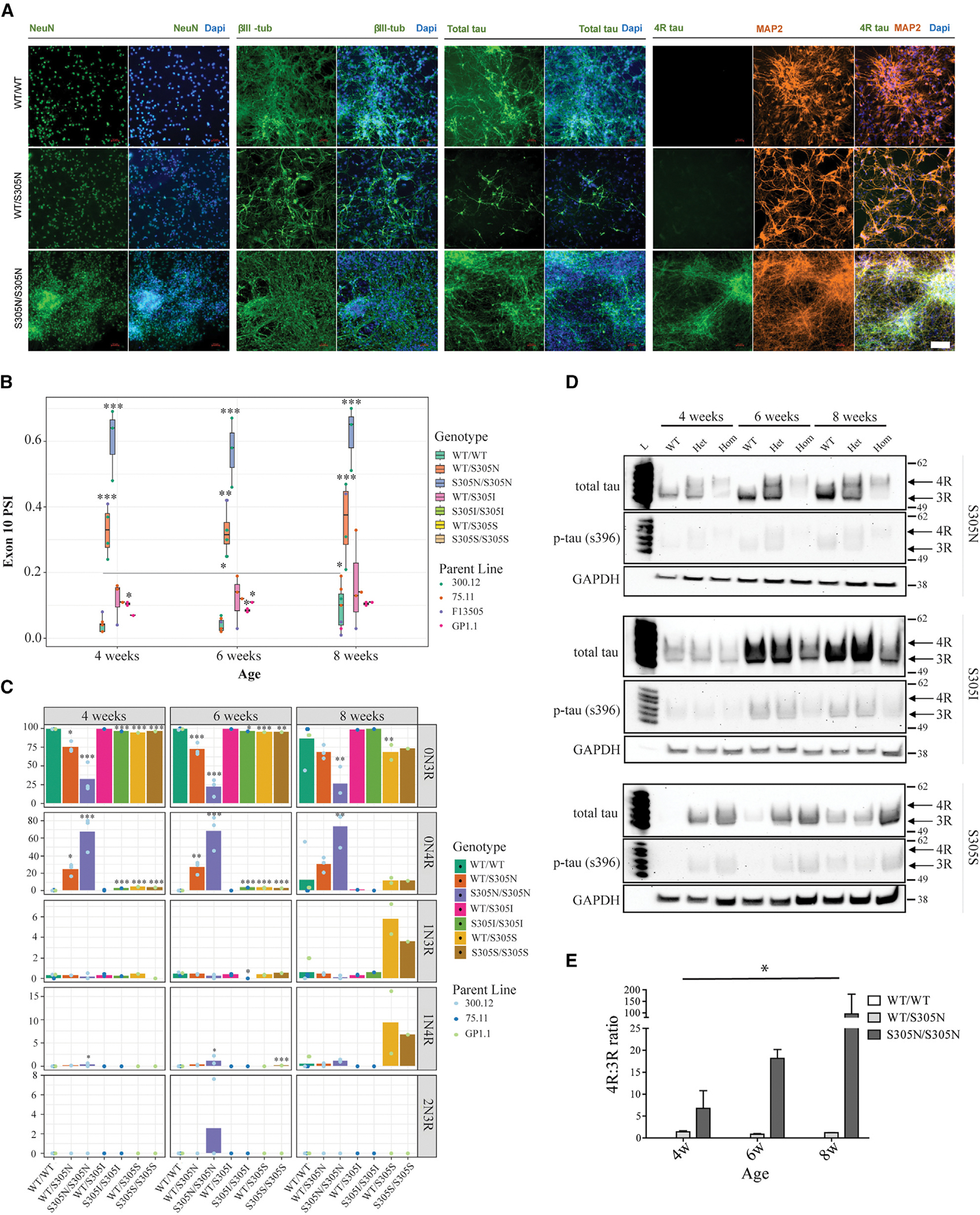
*MAPT* S305 mutations significantly increase exon 10 inclusion in iPSC neurons (A) Representative images of NeuN, βIII-tub, total tau, 4R tau (RD4), and MAP2 staining in an isogenic set of S305N mutation neurons. Scale bar, 100 μm. (B) Percentage spliced in (PSI) values for *MAPT* exon 10 at 4, 6, and 8 weeks in iPSC neurons. Error bars indicate SEM. *n* = 1–7. One-way ANOVA, Tukey *post hoc* testing. (C) Targeted *MAPT* ISOseq for five main neuronally expressed transcripts. Error bars, SEM. *n* = 1–7. Two-way ANOVA, Tukey *post hoc* testing. (D) Representative western blots for total tau and p-tau (S396) in neurons. L, tau ladder. GAPDH was used as a loading control. (E) Quantification of 4R:3R ratio for S305N clones in (D). Error bars indicate SEM. *n* = 3. Two-way repeated-measures ANOVA. Bonferroni *post hoc* testing. *p < 0.05, **p < 0.01, and ***p < 0.001.

We carried out forebrain neuron-directed differentiation from our panel of S305 mutation iPSC lines and isogenic controls and assayed exon 10 inclusion at 4, 6, and 8 weeks of differentiation from neural progenitor cells (NPCs) (Figures 2A–2E and S2A–S2G). We first confirmed that *MAPT* mutation did not alter propensity for neuronal differentiation by assessing maturation marker expression from RNA sequencing (RNA-seq) data (Figure S2A) and by visual inspection of immunofluorescence (Figures 2A, S2B, and S2C). Total *MAPT* expression and exon 10 percentage spliced in (PSI) values were then assessed from RNA-seq data (Figures 2B, S2D, and S2E). Interestingly, while there was a trend toward reduced total *MAPT* expression with age when correcting for parent line (*p* = 0.055), with expression reducing in mutant neurons with time (Figure S2D), this effect was significant only in S305N homozygote lines at 8 weeks of differentiation following *post hoc* testing (*p* = 0.026). In contrast, there was a significant effect of age on exon 10 PSI in WT neurons (F_(5,15)_ = 2.9, *p* = 0.049), with increased inclusion over time, as previously observed^19^ (Figure 2B). Surprisingly, there was no effect of age on exon 10 inclusion in S305S, S305I, or S305N neurons, indicating that these mutations significantly increase 4R inclusion early in differentiation and remain stable over time (Figures 2B and S2E). S305N resulted in the largest increase in exon 10 PSI, with a clear gene dosage effect (mean PSI WT/S305N ~0.3–0.4, S305N/S305N ~0.6, Figure 2B). S305I had the second highest increase in exon 10 PSI, although due to high variability in heterozygous clones, this was not statistically significant (both WT/S305I and S305I/S305I mean PSI ~0.1–0.16). Increased exon 10 inclusion was confirmed by quantitative reverse-transcription PCR (RT-qPCR) in S305I lines compared to isogenic controls across time (Figure S2E). S305S had the mildest effect on exon 10 PSI, which was statistically significant at 4–6 weeks of differentiation, but was equivalent to WT controls by 8 weeks (WT/S305S and S305S/S305S mean PSIs ~0.07–0.1, Figures 2B and S2E). This was surprising, as predictions of RNA stability estimated S305S to be the most destabilizing. Regardless, all mutations influenced exon 10 splicing.

To investigate whether S305 splicing mutations also destabilize regulation of *MAPT* N-terminal splicing, and to characterize full-length isoform expression, we carried out targeted isoform sequencing (ISOseq) (Figure 2C). As expected, iPSC neurons expressed primarily 0N isoforms; however, S305N mutants significantly reduced the proportion of 0N3R at all three time points (WT mean = 86%–99%, WT/S305N = 68%–75%, S305N/S305N = 21.5%–32%) in favor of significantly increased 0N4R (WT = 0.3%–12%, WT/S305N = 24%–30%, S305N/S305N = 67%–73%) (Figure 2C). Similar to the short-read sequencing data, the effect of S305I and S305S mutations on exon 10 inclusion were milder, although both mutations expressed significantly higher levels of 0N4R at 4 and 6 week of differentiation compared to isogenic controls (S305I = 0.7%–2%, S305S = 3.4%–11.5%, Figure 2C). Interestingly, there was a slight increase in the detection of 1N isoforms in S305S and S305N mutations, with a significant increase in 1N3R in S305S heterozygotes at 8 weeks (WT = 0.6%, WT/S305S = 5.8%, S305S/S305S = 2.8%, Figure 2C). S305N mutations showed a trend toward increased 1N4R over time, although this did not reach statistical significance, likely due to very low levels of detection (Figure 2C).

To confirm that we could detect 4R tau protein, we carried out western blots (Figures 2D, 2E, and S2F–S2G). There was a significant effect of both time and genotype on 4R tau expression in S305N neurons (F_(2,18)_ = 3.63, *p* < 0.05). We observed a clear increase in 4R expression in S305N neurons compared to isogenic controls, with homozygous lines expressing almost exclusively 4R tau, compared to 1:1 3R:4R in heterozygous mutants (Figures 2D and 2E). 4R tau was also visualized by immunofluorescence (Figure 2A). In comparison, we were unable to detect 4R tau protein in S305I and S305S mutation lines, likely due to relatively low expression of 4R compared to S305N mutants (Figure 2D). We validated these data in dephosphorylated protein lysates (Figure S3A). In contrast to previous reports for other *MAPT* mutations,^6,8^ we did not observe accumulation of soluble tau over time (Figures 2D and S2F). Instead, there was a trend toward reduced total tau protein in S305N mutants, which did not reach significance (Figures 2D and S2F). This may be due to increased turnover of 4R tau isoforms compared to 3R.^32^ We observed a significantly increased phosphorylated tau (p-tau_s396_)-to-total tau ratio (p-tau/total tau) in S305N homozygous mutants at each time point compared to isogenic controls (Figures 2D and S2G).

### Different *MAPT* S305 mutations have distinct and shared transcriptional effects

In contrast to the synonymous S305S splicing mutation, non-synonymous S305N and S305I mutations both increase 4R *MAPT* expression and alter the tau protein sequence. We hypothesized that expression of mutant 4R tau in these lines may have differential effects on neuronal function compared to expression of WT 4R tau. We therefore carried out gene set enrichment analysis (GSEA) on RNA-seq data to identify the shared and divergent functional effects of each S305 mutation.

**Figure 3.**
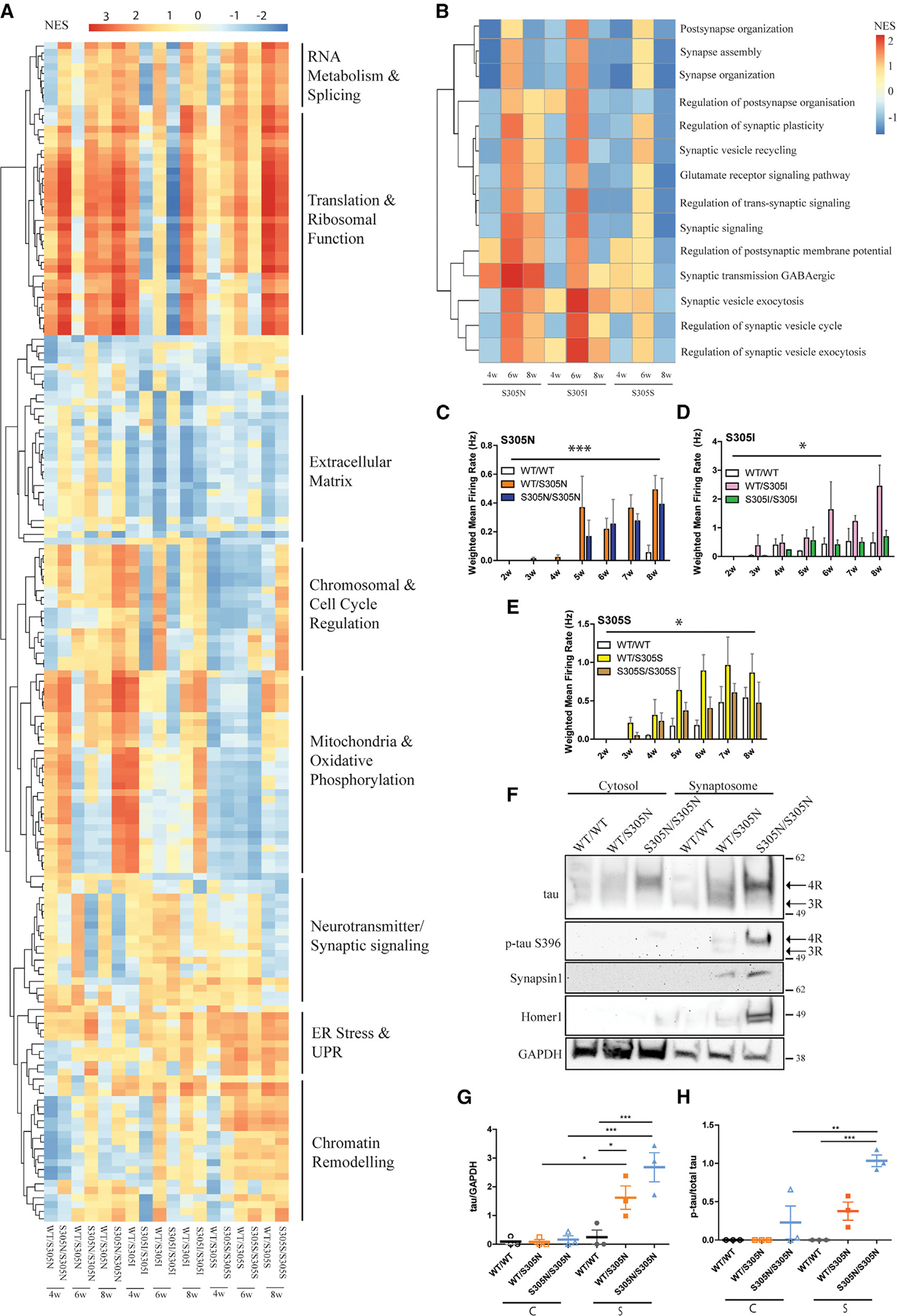
*MAPT* S305 mutations converge on disrupted synaptic function (A) Integration of top 20 significantly enriched GSEA pathways across genotype and age. NES, normalized enrichment score; red, upregulation; blue, downregulation. Pathways underwent unsupervised clustering based on NES across samples. (B) NES from GSEA of synaptic-related pathways for each heterozygous mutation. (C–E) Weighted mean firing rate (Hz) of isogenic sets of (C) S305N, (D) S305I, and (E) S305S iPSC neurons from 3 to 8 weeks of age. Error bars indicate SEM. *n* =3 independent differentiations, averaged from three technical replicates per genotype/clone. Two-way ANOVA, Bonferroni *post hoc* testing. (F) Representative western blot of cytosolic and synaptosome fractions from 6-week S305N neurons and isogenic controls, labeled for total tau and p-tau (S396), with GAPDH loading control. (G and H) Quantification of (F) for (G) total tau and (H) p-tau (S396), normalized to GAPDH in cytosol (C) and synaptosome (S) fractions. Error bars indicate SEM. *n* = 3. One-way ANOVA, Bonferroni *post hoc* testing. *p < 0.05, **p < 0.01, and ***p < 0.001.

When comparing the number of shared pathways by GSEA, we found that heterozygous mutations shared 113 (6.3%) significantly enriched pathways at 4 weeks but became more divergent with age (Figure S3B). In contrast, homozygous mutations retained a consistently similar proportion of overlap across time (19–50 pathways, 1.9%–3.5%) (Figure S3C). Inspection of the top 20 significant pathways for each gene dosage at each time point revealed pathways most strongly impacted by each mutation (Figure 3A; Table S2). S305N was associated with upregulation of pre- and post-synaptic components, including GABAergic synaptic transmission, as well as early upregulation of translation and ribosomal pathways at 4 weeks of differentiation, which was maintained with age. Other pathways upregulated across differentiation were involved in cell-cycle regulation, endoplasmic reticulum (ER) stress, and oxidative phosphorylation (Figure 3A; Table S2).

S305I showed a more complex relationship between heterozygous and homozygous mutation carriers over the course of differentiation but exhibited similar pathway enrichment by 8 weeks (Figure 3A; Table S2). This mutation was associated with progressive upregulation of oxidative phosphorylation as well as downregulation of extracellular matrix organization. Similar to S305N lines, we observed upregulation of ribosomal and ER stress pathways, as well as changes to RNA metabolism (Figure 3A; Table S2).

Finally, S305S was also associated with upregulation of ribosomal and translational regulation pathways, as well as increased ER and unfolded protein response (UPR) pathways. Specific to S305S was an increase in heat shock response and nuclear export, as well as increased histone methylation (Figure 3A; Table S2).

### *MAPT* S305 mutations accelerate synaptic maturation and neuronal hyperexcitability

When comparing the top GSEA pathways across all S305 mutations, we observed dysregulation of pathways associated with neurotransmitter signaling and GABAergic receptor signaling (Figures 3A and S3D). Notably, glutamate-related signaling pathways were not in the top 20 GSEA results from any mutation. This was surprising, as we had previously characterized upregulation of glutamatergic receptor genes in *MAPT* V337M mutation neurons,^6^ and other *MAPT* mutation iPSC neurons exhibit electrophysiological hyperexcitability.^7^ For further inspection of synaptic disruption, we focused on heterozygote mutant carriers only, as two parent lines contributed to these signals compared to only one parental donor for the homozygote edits. Here, we observed that disrupted synaptic signaling pathways showed genotype-specific and temporally regulated dysregulation (Figure 3B). This pattern was consistent with the proportion of exon 10 expressed by each mutation, with more severe neuronal synaptic dysregulation correlating with increased exon 10/4R tau expression.

Surprisingly, many synaptic pathways were downregulated across all three mutations at 4 weeks of differentiation, followed by significant upregulation at 6 weeks and a subsequent decline at 8 weeks (Figure 3B; Table S3). However, “synaptic transmission GABAergic” was significantly increased (NES = 1.66, adjusted *p* = 0.02) early in S305N mutants, which was maintained over time, whereas glutamate-related pathways were unaffected until 6 weeks (Figure 3B; Table S3). In contrast, S305I mutation neurons showed no significant effect on GABAergic signaling at any time point, but had significant upregulation of exocytosis- and vesicle transport-related pathways at 6 weeks (Figure 3B; Table S3). S305S mutation neurons exhibited the mildest effect on synaptic function. While there was upregulation of multiple pathways at 6 weeks, similar to S305N and S305I mutations, this did not reach significance. However, the S305S mutation resulted in the largest downregulation of these pathways at 8 weeks, including significantly reduced “glutamate receptor signaling pathway” (NES = −1.59, adjusted *p* = 0.03) and “synaptic signaling” (NES = −1.58, adjusted *p* = 0.04). These data indicate that disrupted synaptic signaling is a convergent feature of all S305 mutations, but each mutation uniquely impacts these mechanisms.

To determine whether altered gene expression pathways translated to functional changes in neuronal firing, we carried out multi-electrode array (MEA) analysis. All three mutations exhibited an increased number of spikes and significantly increased weighted mean firing rate compared to isogenic controls from as early as 3 weeks of differentiation (Figures 3C–3E and S4A), consistent with previous reports of early hyperexcitability in *MAPT* mutation neurons.^7^ Interestingly, there was no mutation dosage effect in S305N neurons, despite differences in 4R protein expression, although both WT/S305N and S305N/S305N were more active than isogenic controls from 3 weeks (Figures 3C and S4A). S305I neurons also showed increased firing from as early as 3 weeks, although heterozygote mutants were more active than homozygotes (Figures 3D and S4A), consistent with the GSEA (Figure S3D). Finally, S305S mutants exhibited the same hyperexcitability from 3 weeks, despite lower levels of 4R tau compared to S305N mutants (Figures 3E and S4A). The isogenic control line showed a similar level of spontaneous activity by 7–8 weeks, consistent with the pattern of exon 10 inclusion we observed across this time frame (Figures 2B–2D).

Given the transcriptomic and functional effects on synaptic firing observed across S305 mutations, and that mutant tau protein has been reported to accumulate in and interact with components of the synapse,^33,34^ we isolated synaptosomes from S305 mutation iPSC neurons and isogenic controls to assess tau accumulation and synaptic maturity (Figures 3F–3H and S4B–S4F). Consistent with reports from mouse and human brain,^35–37^ total tau and p-tau_s396_ preferentially accumulated in S305N and S305I synaptosomes compared to the cytosol and in comparison to isogenic controls, although S305S lines had similar total tau and p-tau_s396_ levels compared to controls (Figures 3F–3H and S4C–S4F). We also saw increased presence of synaptic markers Homer1 and Synapsin1 in S305I and S305N synaptosome preparations compared to isogenic controls and a trend toward increased synaptic marker expression in S305S synaptosomes (Figures 3F and S4B–S4F). This suggests that synapses may be more readily formed and/or matured in the presence of 4R tau, which may underlie the increased neuronal firing observed by MEA.

### Mitochondrial function is differentially affected by WT and mutant 4R tau

One of the most interesting differences between S305 mutation lines by GSEA was the effect on mitochondria-related pathways, such as “oxidative phosphorylation” and “electron transport chain” (Figure 3A; Table S2). Curiously, these pathways were upregulated early in both S305I and S305N neurons, but downregulated or unaffected in S305S neurons, indicating a differential effect between increased WT 4R tau and mutant 4R tau.

**Figure 4.**
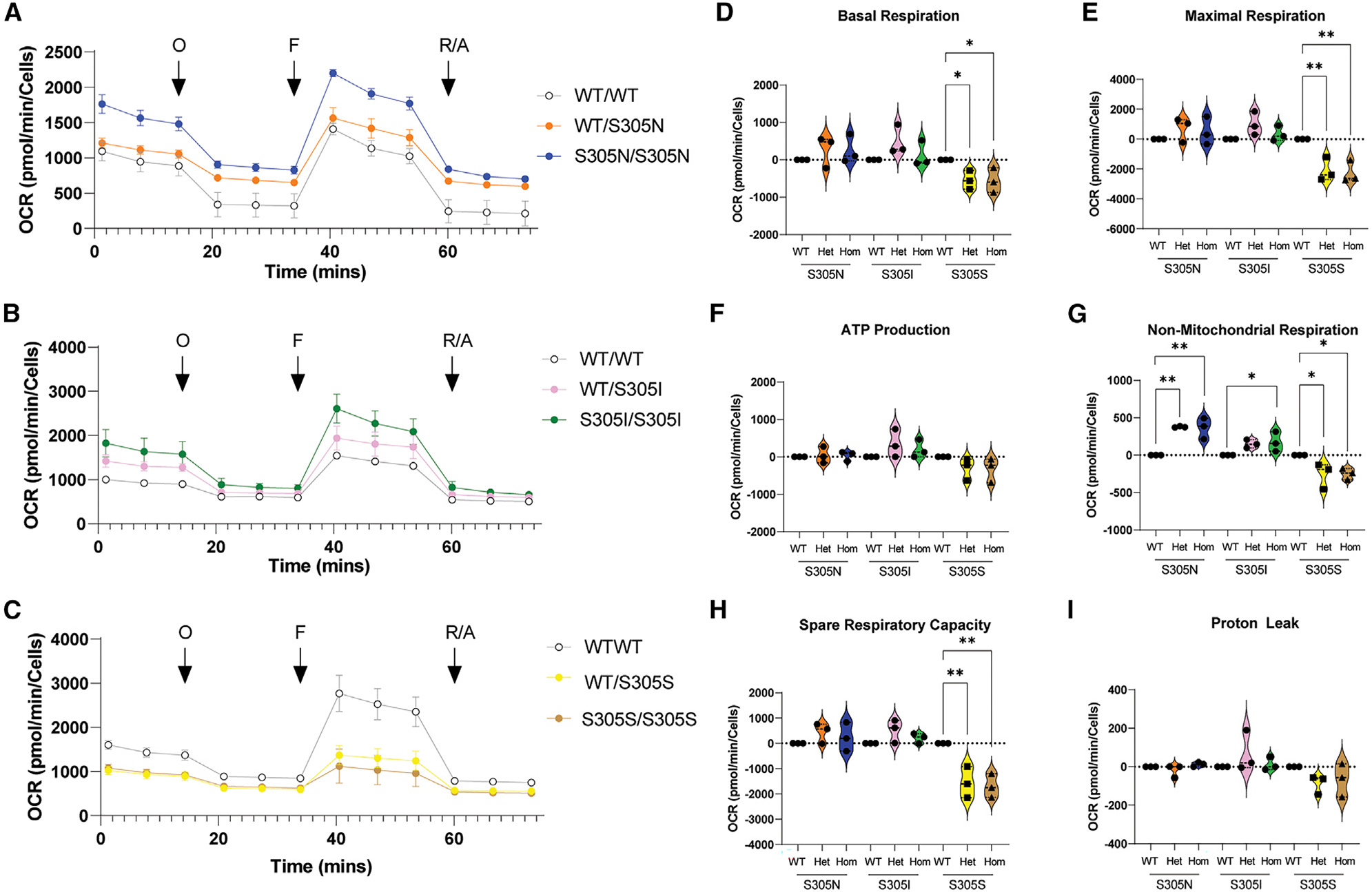
Mitochondrial bioenergetics are altered in *MAPT* S305 mutation neurons (A–C) Representative plots of oxygen consumption rate (OCR, pmol/min corrected for number of cells) in isogenic sets of (A) S305N, (B) S305I, and (C) S305S neurons at 6 weeks: output from the Seahorse mitochondrial stress test. Arrows indicate time of treatment with stressors; O, oligomycin; F, FCCP; RA, rotenone/antimycin-A. Error bars indicate SEM. *n* = 3. (D–I) Quantification of mitochondrial (D) basal respiration, (E) maximal respiration, (F) ATP production, (G) non-mitochondrial respiration, (H) spare respiratory capacity, and (I) proton leak relative to isogenic controls in *MAPT* S305 mutation neurons at 6 weeks. Error bars indicate SEM. *n* = 3 independent differentiations, averaged from three technical replicates. One-way ANOVA, Fisher’s least significant difference (LSD) *post hoc* testing. *p < 0.05, **p < 0.01, and ***p < 0.001.

To functionally examine whether S305 mutations disrupt mitochondrial function, we carried out Seahorse mitochondrial stress tests on 6-week-old neurons (Figures 4A–4I). Consistent with pathway analyses, we observed a trend toward increased basal respiration in both S305N and S305I neurons compared to isogenic controls (Figure 4D), indicating more oxygen consumption was required to meet neuronal energy demands, but significant downregulation in S305S neurons (Figure 4D), suggesting reduced energy demand. The same trend was present in the S305N and S305I neurons for both maximal respiration and spare respiratory capacity (Figures 4E and 4H), which may be reflective of their increased potential for hyperexcitability and therefore increased bioenergetic demand. Consistent with the RNA-seq data, these measurements were significantly reduced in S305S neurons (Figure 4E and 4H), potentially indicating reduced mitochondrial capacity for oxidative phosphorylation. There were no significant differences in either ATP production or proton leak between genotypes, indicating that mitochondrial membranes were intact and sufficient ATP was being generated to meet energy demands (Figures 4F and 4I). Interestingly, non-mitochondrial respiration was significantly increased in both S305N and S305I neurons (Figure 4G). High non-mitochondrial respiration has been associated with increased energy production via glycolysis, but has also been linked with increased inflammation and oxidative stress,^38,39^ both processes that have been associated with tauopathy.^40–42^ In contrast, non-mitochondrial respiration was significantly reduced in S305S neurons (Figure 4G), indicating that the majority of oxygen consumption in these cells was being used for mitochondrial respiration and that cellular bioenergetics may be impaired.

### *MAPT* splicing is altered in S305 mutation astrocytes and is distinct from neuronal splicing regulation

Astrocytes express low levels of *MAPT*, which affects intrinsic astrocytic functions, including altered glutamate processing, neuronal support, and amyloid-β-induced synaptotoxicity.^43–45^ We therefore differentiated our S305 mutation panel into astrocytes and assessed the effects of these mutations on astrocytic *MAPT* expression and splicing.

**Figure 5.**
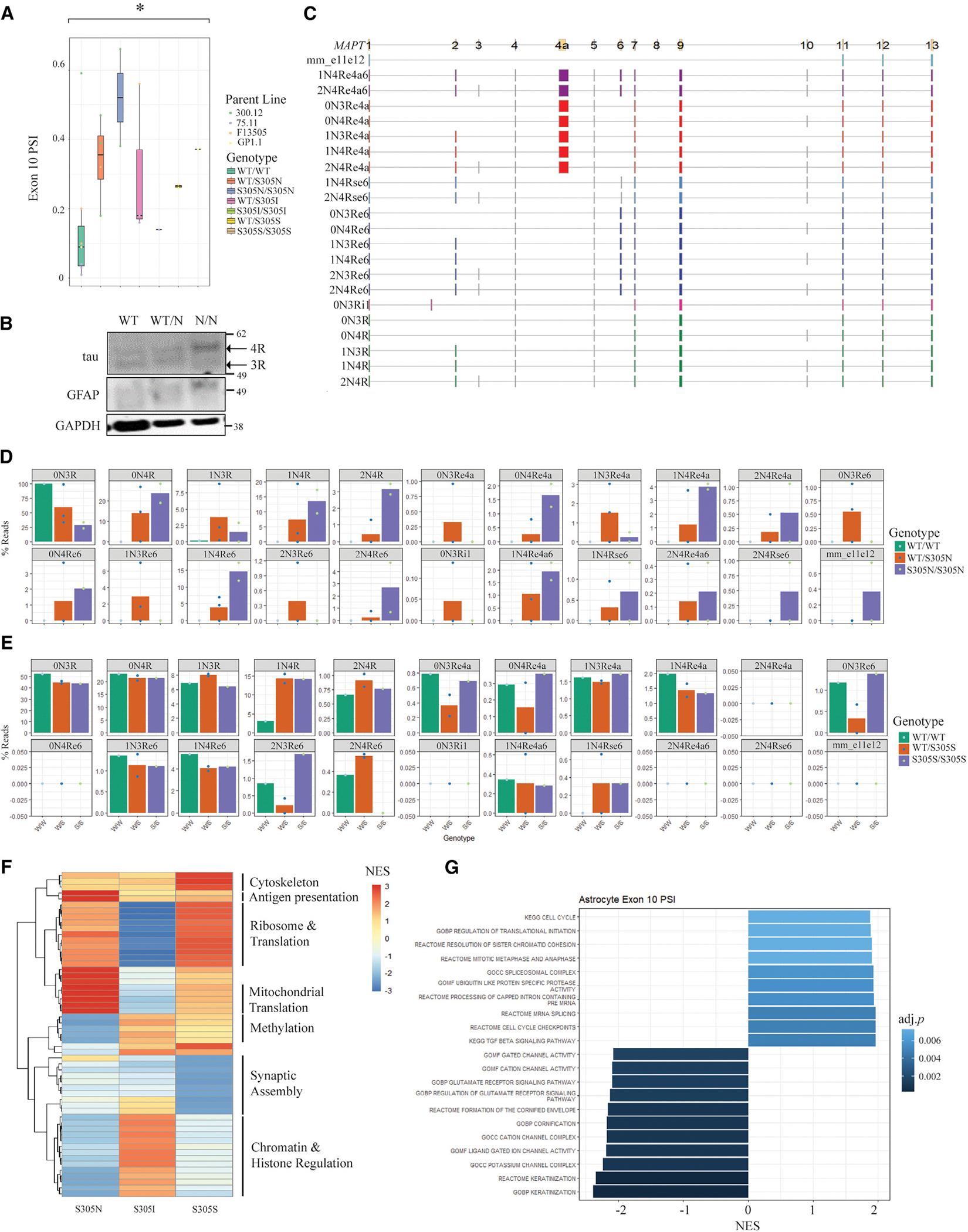
*MAPT* S305 astrocytes have a distinct *MAPT* splicing profile (A) *MAPT* exon 10 percentage spliced in (PSI) values derived from RNA-seq data in astrocytes. Error bars indicate SEM. *n* = 1–7. One-way ANOVA. (B) Representative western blot of tau expression in S305N astrocytes. GFAP was used as a loading control. (C) Schematic of different *MAPT* transcripts observed in iPSC astrocytes. “mm_e11e12,” most exons missing, except for 11 and 12; “e4a,” inclusion of exon 4a; “6” or “e6,” inclusion of exon 6; “se6,” inclusion of small variant of exon 6; and “i1,” retention of intron 1. Transcripts are colored by splice pattern (canonical, green; intron retention, pink; exon 6, dark blue; small exon 6, light blue; exon 4a, red; exons 4a and 6, purple; mostly missing exons, pale blue). (D and E) Percentage of reads mapping to each transcript identified from targeted ISOseq data in (D) S305N and (E) S305S mutation astrocytes. Error bars indicate SEM. *n* = 1–3. One way ANOVA. (F) Normalized enrichment score (NES) for top 10 significantly enriched up- and downregulated GSEA pathways shared across S305 mutation astrocytes. Blue, downregulation; red, upregulation. (G) NES for significantly enriched GSEA pathways calculated as a function of exon 10 PSI in S305 mutation astrocytes. Shading represents adjusted enrichment *p* value (adj.*p*).

*MAPT* expression in astrocytes was around 50-fold lower than in neurons (neurons ~10,000 reads per sample, astrocytes ~200 reads per sample). Similar to the neurons, there was no significant effect of mutation on total *MAPT* expression after correction for donor; however, there was a significant effect on exon 10 PSI (F_9,10_ = 3.6, *p* < 0.05) (Figure 5A). Interestingly, S305I and S305S mutations had a larger impact on exon 10 PSI in astrocytes compared to neurons, with a PSI of ~20%–30% in mutant astrocytes and 5%–15% in mutant neurons (Figures 2 and 5A). S305N mutations exhibited the most severe effect on splicing, with a clear mutation-dosage effect (Figure 5A), which was validated by western blot (Figure 5B).

Given the differences in exon 10 inclusion compared to neurons, we carried out targeted *MAPT* ISOseq to examine differences in full-length transcripts between S305 mutations (Figures 5C–5E). Technical issues resulted in very poor read depth for 75.11-derived and F13505-derived lines; therefore, we focused these analyses on the most severe 300.12 (S305N) and milder GP1.1 (S305S) lines. We identified a wider diversity of *MAPT* transcripts in astrocyte cultures compared to neurons (Figure 5C). We detected both 1N and 2N transcripts, as well as exons 6 and 4a, which define the “Big Tau” isoform present in the peripheral nervous system (PNS)^46^ (Figure 5C). For the S305N isogenic corrected control line, only 0N3R and 1N3R were detected, whereas heterozygous and homozygous mutants expressed multiple other, primarily 4R, transcripts (Figure 5D). Interestingly, S305S mutation astrocytes did not have significantly higher expression of 0N4R compared to their isogenic controls, but instead were enriched for 1N4R (Figure 5E).

As these comparisons have been conducted in only one parent line per mutation, with a single isogenic corrected control, and there was significant variability between the two isogenic corrected controls, it is not possible to confidently state the effect of S305 splicing mutations on the splicing of other exons in astrocytes. However, these data support the increased inclusion of exon 10 in S305 mutation astrocytes and demonstrate that astrocytes splice *MAPT* in a manner that is distinct from neurons.

### iPSC astrocytes demonstrate cell-autonomous effects of mutant *MAPT* expression

To characterize the effect of S305 mutations on astrocyte function, we carried out RNA-seq and GSEAs on differentially expressed genes within each mutation, while controlling for mutation dosage (Figure 5F; Table S4). All three mutations resulted in upregulation of cytoskeleton- and antigen-presentation pathways (Figure 5F; Table S4), suggesting that mutant *MAPT* expression in astrocytes may be altering cellular morphology and inducing an inflammatory phenotype. All three mutations were associated with significant downregulation of glutamate and neurotransmitter signaling pathways (Figure 5F; Table S4), consistent with previous reports that *MAPT* mutation astrocytes are impaired in glutamate processing and clearance.^43^

In concordance with our observations in iPSC neurons, we observed multiple disparate effects of S305 mutation in the astrocytes (Figure 5F; Table S4). For example, in contrast to their neuronal counterparts, S305I astrocytes were associated with significant downregulation of ribosomal and translation-regulation pathways, which were upregulated in neurons (Figure 3A), while the same pathways were significantly upregulated in S305N and S305S astrocytes (Figure 5F). These data suggest that specific *MAPT* mutations are likely to have disparate effects on similar cellular processes across different cell types independent of exon 10 inclusion.

We then repeated the GSEA using exon 10 PSI values for each line as the defining variable to assess the effect of exon 10 inclusion on astrocyte gene expression (Figure 5G; Table S5). We found that increasing exon 10 expression was associated with upregulation of multiple cell-cycle and chromosomal regulation pathways (Figure 5G), consistent with previous findings of abnormal cell-cycle reentry in a humanized tau mouse model.^47^ In contrast, increased *MAPT* exon 10 PSI was significantly associated with downregulation of glutamate receptor signaling and ion channel activity (Figure 5G), indicating that in astrocytes, increased exon 10 inclusion may be interfering with neuronal-glial cellular signaling and communication.

### Tau internalization and inflammation are modulated by *MAPT* S305 mutations in astrocytes

**Figure 6.**
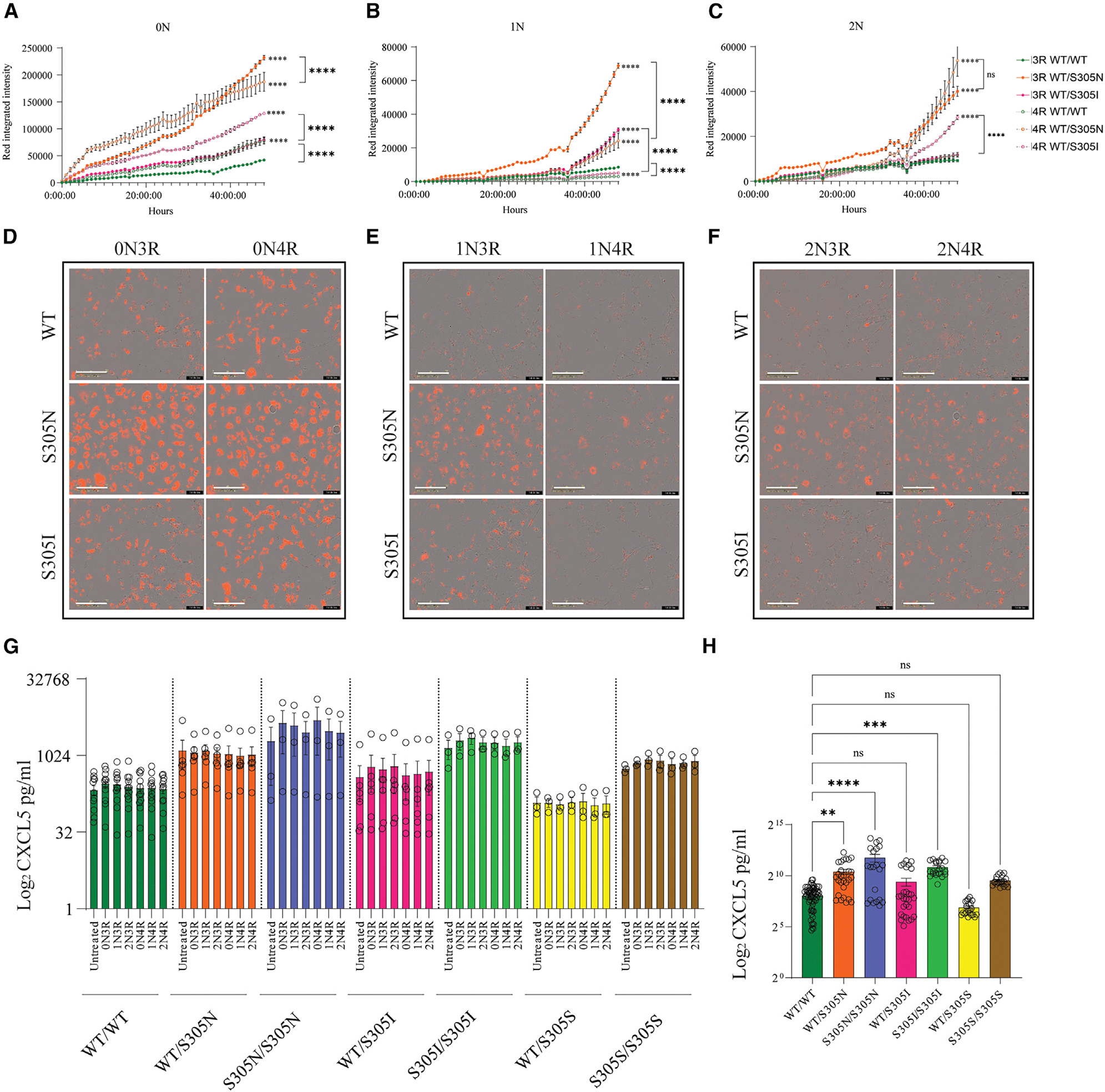
*MAPT* S305 astrocytes rapidly internalize exogenous tau (A–C) Representative traces of integrated fluorescence intensity of pHrodo Red in S305 mutation astrocytes and isogenic controls treated with labeled (A) 0N3R and 0N4R, (B) 1N3R and 1N4R, and (C) 2N3R and 2N4R tau over 48 h. Error bars indicate SEM. *n* = 4; each point is an average of three fields of view per well. Linear regression. (D–F) Representative images of S305 mutation astrocytes and controls at 48 h following treatment with pHrodo-labeled (D) 0N, (E) 1N, and (F) 2N tau, quantified in (A–C). Scale bars, 200 μm. (G) Log_2_ CXCL5 concentration (pg/mL) in astrocyte culture medium, corrected for cellular density, following treatment with exogenous tau. Error bars indicate SEM. *n* = 3–12; each point represents averaging of three technical replicates. Two-way ANOVA, Dunnett’s multiple comparison tests. (H) Log_2_ CXCL5 concentration (pg/mL) in astrocyte culture medium, corrected for cellular density, pooled across treatment conditions. Error bars indicate SEM. *n* = 21–70. One-way ANOVA, Dunnett’s multiple comparison tests. ns, not significant; *p < 0.05, **p < 0.01, ***p < 0.001, and ****p < 0.0001.

Accumulation of tau in astrocytic tufts is a neuropathological feature of PSP and is commonly observed in FTD brain.^48^ However, as *MAPT* expression is very low in these cells, the source of accumulated tau is thought to be exogenous to astrocytes. As we observed dysregulation of pathways associated with endocytosis in S305 mutant astrocytes, we tested whether increased *MAPT* exon 10 inclusion may influence the internalization of tau. We therefore incubated S305 mutation and isogenic control astrocytes with six different isoforms of monomeric recombinant tau, labeled with pHrodo Red, and tracked tau internalization over 48 h (Figures 6A–6F and S5A–S5C).

We observed a significant effect of both genotype and isoform on the rate of tau internalization (Figures 6A–6F and S5A–S5C) for all S305 mutations. Specifically, 0N isoforms were internalized faster than 1N isoforms, while 2N isoform internalization was inefficient across most genotypes (Figures 6A–6F and S5A–S5C). The preferential and specific uptake of 0N and 1N tau was consistent with our previous report that 2N tau was not observed in astrocyte pathologies in either PSP or Alzheimer’s disease (AD) brain.^49^ The relationship between 3R and 4R isoforms was more complex; there was no consistent effect of 3R/4R tau for 0N or 2N isoforms across all three parent lines. However, 1N3R was consistently internalized at a significantly faster rate than 1N4R tau across all mutations and parent lines (Figures 6A–6F and S5A–S5C), indicating that both N- and C-terminal splicing of *MAPT* affect its uptake. For all parent lines, S305 mutations showed significantly increased tau internalization compared to their isogenic control (Figures 6A–6F and S5A–S5C). Interestingly, comparison of S305N and S305I mutations on the same genetic background (F13505) revealed an association between the rate of tau internalization and the extent of *MAPT* exon 10 expression; tau uptake was faster in S305N mutants than in S305I (Figures 6A–6F), suggesting that 4R tau may directly influence astrocyte protein internalization.

As our gene expression data indicated a potential effect of *MAPT* S305 mutations on astrocytic basal inflammatory state, and inflammation has been described in PSP brain,^50,51^ we hypothesized that exogenous tau exposure could induce an inflammatory response in astrocytes that is modulated by S305 mutations. We therefore tested this hypothesis by conducting ELISAs for CXCL5 on the supernatant from tau-treated astrocytes (Figures 6G and 6H). Contrary to expectations, we did not observe any effect of tau treatment on the release of CXCL5 for any mutation (Figure 6G), indicating that exogenous tau exposure did not induce an inflammatory response. However, we did observe significantly increased CXCL5 at baseline in S305N and S305I mutant astrocytes (Figure 6H), indicative of an altered inflammatory state in these cells that could influence downstream function.

## DISCUSSION

Given the importance of 4R tau in both healthy and diseased brain, low levels of 4R tau expression in iPSC cultures have inhibited complete and accurate modeling of tauopathy. This has been partially addressed by utilizing iPSC lines carrying *MAPT* mutations in constitutively expressed exons, which are present in the dominantly expressed 3R isoform.^6,8,11,14,15,31^ In addition, iPSC neurons carrying the *MAPT* P301L mutation, which is in exon 10, still display tauopathy-relevant phenotypes despite very low 4R tau expression,^7,11^ indicating that even low-level exon 10 inclusion substantially impacts cellular function. Consistent with this assertion, 4R tau more strongly stabilizes microtubules compared to 3R, reduces cellular size, influences phosphorylation, and modulates inflammatory responses.^52–56^ It is therefore important to model and manipulate the functional effects of 4R tau in a human context to understand its contribution to pathogenic mechanisms across different cell types and to facilitate screening of therapeutics in a more representative model of the human cellular environment.

For this purpose, we developed an isogenic panel of iPSC lines, derived from four different donors, including heterozygote and homozygote *MAPT* S305N, S305I, and S305S splicing mutations and paired isogenic corrected controls, totaling 26 clones. We found that S305N had the most severe effect on exon 10 inclusion, resulting in 70% 4R transcripts from as early as 4 weeks of neuronal differentiation. Indeed, S305N was identified as one of the most destabilizing modifications possible in the hairpin-loop region.^28^ S305I mutants were second in exon 10 inclusion; however, substantial variability in heterozygous mutants possibly reflected donor line variability, as the healthy control iPSC line (F13505) into which we introduced the S305I mutation resulted in less exon 10 inclusion compared to the patient-derived clones (75.11). This suggests that interaction with individual genetic backgrounds may influence the severity of S305I, as has been suggested for other pathogenic mutations.^57,58^ Regardless, we observed consistent transcriptomic and functional effects of this mutation across donor lines, despite variability in 4R tau expression.

The synonymous S305S mutation had the least severe and minimal influence on exon 10 inclusion, although it was sufficient to induce functional and transcriptomic changes comparable to those of S305N and S305I. This reduced severity reflects the clinical syndrome, in which age of onset in affected carriers is into the 40s and 50s,^26,27^ compared with an age of onset of 30–40 and subsequent death within 2 years for currently reported S305N and S305I carriers.^24,25^ The milder effect of S305S parallels the characterization of *MAPT* IVS10+16 mutations, which also destabilize the exon 10-intron 10 hairpin loop without introducing an amino acid change and have a similar age of onset.^59^ However, unlike IVS10+16 iPSC neurons, which show progressive exon 10 inclusion over several months of differentiation,^19,20^ S305S as well as S305N and S305I mutations had an early and sustained influence on exon 10 splicing. Longer culture maintenance would be required to confirm whether 4R tau expression changes over a prolonged period across all three S305 mutations. Early aberrant expression of 4R tau has implications for considering susceptibility for neurodegenerative disorders and the developmental impact of pathogenic mutations. Higher 3R tau expression in human fetal brain is more permissive of the synaptic plasticity and remodeling necessary for healthy brain development,^4,60^ due to its reduced ability to stabilize microtubules. Indeed, overexpression of 4R tau in SH-SY5Y cells promoted neuronal maturation when 3R tau did not.^61^ Expression of 4R tau too early in neuronal development may therefore have fundamental effects on cellular function, with downstream consequences for disease susceptibility later in life. This potential mechanism may be most apparent in S305S neurons, as there remained significant transcriptomic changes associated with the mutation at 8 weeks of differentiation, even though the proportion of exon 10 in isogenic corrected controls was comparable to the mutants by this time point.

Consistent with other *MAPT* mutation models, we observed dysregulation of synaptic signaling in S305 mutation neurons. Curiously, while all mutations converged in synaptic dysfunction, the exact mechanism by which synaptic pathways were affected differed across mutation. Interestingly, glutamatergic signaling was typically downregulated at 4 weeks of differentiation across all three mutations and at 8 weeks in S305S mutation neurons. This was surprising, as we and others have consistently observed upregulation of glutamatergic signaling pathways in iPSC models with different *MAPT* mutations, as well as increased neuronal firing and hyperexcitability.^6,7,17,62^ However, we still observed early neuronal activity and synaptic maturation in S305 mutants, consistent with observations from V337M, N279K, and P301L *MAPT* mutation iPSC neurons.^7,62^ A recent electrophysiological study of IVS10+16 mutation neurons suggested that increased 4R tau caused impaired neuronal excitability,^17^ although these studies were conducted much later in differentiation than ours. This discrepancy may be indicative of a progressive phenotype that switches from hyperexcitability to impairment with age, as has been previously suggested,^6,7^ and is supported by our transcriptomic data from S305S neurons at 8 weeks of differentiation. Alternatively, downregulation of these pathways in our data may be indicative of a compensatory response to increased synaptic maturation and neuronal activity. The reciprocal relationship we observed between misregulation of numerous synaptic signaling pathways, coupled with increased electrophysiological activity in S305 mutants over time, suggests that early neuronal hyperexcitability commonly observed in different *MAPT* mutation models may occur via different complex mechanisms that ultimately converge on alteration of the excitatory/inhibitory balance. Similar to observations in mouse brain,^63,64^ we observe increased localization of tau and phosphorylated tau to synaptosomes in S305 mutant neurons, where it has the potential to alter or disrupt synaptic function via modulation of pre-synaptic vesicle recycling, endocytosis impairment, and post-synaptic interaction with Fyn.^65–67^

Although S305N mutants tended to show the most extreme phenotypes and largest gene expression changes compared to S305I and S305S, it is unclear how much of this effect is attributable to the amino acid change or to high 4R tau. The divergent mechanistic changes between missense (S305N/S305I) and synonymous (S305S) mutations provide an indication of WT 4R vs. mutant 4R effects. One of the most apparent differences consistently observed between missense and synonymous mutations was in mitochondrial bioenergetics. Recently, WT tau (2N4R) was demonstrated to directly interact with different components of the mitochondrial membrane, but specific protein interactions changed with the introduction of P301L or V337M *MAPT* mutations, such that they altered mitochondrial function.^34^ It is therefore plausible that the S305N/I amino acid changes modify the tau mitochondrial interactome, resulting in divergent functional effects on bioenergetics compared to a WT 4R protein. Our data from S305S neurons suggest there are likely to be additional differences between 3R and 4R tau in their interaction with the mitochondrial membrane, although the specific effect of isoform has not yet been explored. However, general suppression of mitochondrial bioenergetics in these cells may indicate an inhibition of mitochondrial function caused by 4R tau, rendering neurons less able to cope with alterations in energetic demands.

Notably, the most significant effect of missense S305 mutations was on non-mitochondrial respiration and could reflect the use of glycolysis as an energy source rather than, or in addition to, mitochondrial oxidative phosphorylation. The switch from glycolysis in NPCs to oxidative phosphorylation in neurons has been well documented^34,68^ and would suggest that energy production in S305N and S305I mutant neurons is not undergoing this switch efficiently. Alternatively, increased glycolysis may reflect response to an inflammatory state^69^ to deal with increased energetic demand of cellular stress. This increase in non-mitochondrial respiration may explain the susceptibility to oxidative stress and excitotoxicity observed in other *MAPT* mutation models,^6,8^ as neurons are unable to fully utilize oxidative phosphorylation to cope with additional energy demands. However, a trend toward increased basal and maximal respiration and significant enrichment of oxidative phosphorylation gene expression pathways in these cells is not fully consistent with this hypothesis but could reflect a compensatory response to inefficient ATP production, which was unchanged in these neurons.

Consistent with the assertion that missense S305 mutations may be inducing an inflammatory state in neurons, whereas the synonymous S305S does not, we observed a similar pattern in S305 mutation astrocytes. Both S305N and S305I mutations were associated with increased basal CXCL5, a cytokine associated with aged astrocytes and the promotion of neutrophil chemotaxis in response to inflammatory stimuli.^70,71^ Increased CXCL5 detection in postmortem cerebrospinal fluid (CSF) has recently been observed in cases of chronic traumatic encephalopathy (CTE)^72^ and AD-related mild cognitive impairment (AD-MCI),^73^ supporting the relevance of this cytokine to tauopathy pathophysiology, although the mechanism by which astrocytic endogenous tau may promote CXCL5 release is unclear.

Recently, more attention has been given to the role of astrocytes in tauopathy, as well as the recognition that even very low levels of *MAPT* expression in these cells has potential for cell-autonomous and non-cell-autonomous effects.^43,74,75^ Like recent studies in IVS10+16 iPSC astrocytes,^43^ we also observed downregulation of pathways associated with glutamate and neurotransmitter signaling, consistent with the hypothesis that *MAPT* mutation astrocytic dysfunction contributes to neuronal excitotoxicity by being less able to clear excess glutamate away from the synaptic cleft.^76^ Others have also noted that iPSC astrocytes splice *MAPT* differently from neurons, and the effect of the IVS10+16 mutation on exon 10 inclusion is exacerbated in astrocytes.^75^ We observe the same phenomenon in S305 mutation astrocytes, although our ISOseq data also indicate that *MAPT* splicing regulation is generally different in astrocytes compared to neurons across the full length of the gene. Specifically, we observe a wider diversity of transcripts and the expression of longer transcripts in astrocytes compared to neurons, with increased inclusion of exons 2, 3, and 10, but also exons 6 and 4a, which are not typically considered to be expressed in human brain. Exon 4a is considered to be restricted to the PNS and projecting neurons and absent from the CNS.^46,77^ However, low-level astrocyte-specific expression may have precluded its detection to date. Very little is known about the function of exon 4a, although it may provide additional stabilization to microtubules that facilitates longer-range axonal projections required in the PNS.^46^ A potential function for exon 4a inclusion in astrocytes is unclear. It remains to be seen what the functional impact of astrocytic *MAPT* splicing may be, what the effect of differential isoform usage is in these cells, and the relationship to disease pathogenesis. It should also be noted that we have made these observations only in iPSC-derived astrocytes; therefore, confirmation of altered *MAPT* splicing in human brain-derived astroglia is required to confidently conclude that astrocytes process and splice *MAPT* in this manner.

Finally, as tau accumulation in astrocytes is a characteristic neuropathological feature of several primary tauopathies, we explored whether *MAPT* S305 mutations influenced astrocytic internalization of exogenous tau protein. Internalization of monomeric tau has been shown in rodent astrocytes,^78^ but we now demonstrate in a human system that *MAPT* mutations influence the uptake of tau in a manner correlated with the proportion of exon 10 expression, therefore implicating 4R tau in astrocyte protein internalization capabilities. Surprisingly, we saw a significant effect of tau isoform on the rate of astrocytic internalization, with 0N isoforms most rapidly being processed and 2N isoforms largely remaining unphagocytosed. This pattern of internalization is consistent with what has been observed in human brain pathology: tau pathology in AD and PSP brain primarily consists of 0N and 1N isoforms, whereas 2N isoforms were not present within glia.^49^ These differences may therefore reflect the differential ability of exogenous N-terminal isoforms to enter cells and subsequently accumulate. We did not observe an inflammatory response to treatment with exogenous tau protein; however, this may be due to our use of monomeric recombinant tau without post-translational modification. It therefore remains to be seen how S305 mutation astrocytes respond to oligomeric or hyperphosphorylated protein. Alternatively, as an inflammatory state can promote phagocytosis,^79^ and S305 mutation lines with increased tau internalization had increased CXCL5 release at baseline, it is possible that inflammation occurs prior to, and subsequently exacerbates, tau internalization and accumulation in astrocytes. Indeed, glial inflammation has been found to exacerbate tau pathology in mouse models.^80,81^ In addition, as protein sequence and length influence astrocytic uptake of different tau isoforms, it remains to be seen whether S305 mutations differentially influence the internalization of mutant protein or other substrates that could affect downstream cellular function.

In conclusion, we have developed a valuable panel of isogenic *MAPT* S305 mutation iPSC lines, which express unprecedented levels of 4R tau in iPSC-derived neurons and astrocytes. These lines recapitulate previously observed neuronal phenotypes, such as hyperexcitability and alterations in synaptic function. They also highlight functional differences between missense *MAPT* mutations and pathological WT 4R tau expression in pathways such as mitochondrial bioenergetics, which may have implications for how we understand sporadic compared to familial disease pathogenesis. We also highlight the functional importance of *MAPT* expression in astrocytes and implicate 4R tau in the regulation of inflammatory and phagocytic processes. We believe that these lines will be valuable to tauopathy researchers, enabling a more complete understanding of the pathogenic mechanisms underlying 4R tauopathies across different cell types.

### Limitations of the study

We have generated a unique panel of *MAPT* S305 mutation iPSC lines and have demonstrated multiple phenotypes related to tau pathogenesis in both neurons and astrocytes. However, several outstanding questions remain. While S305N had a severe effect on *MAPT* splicing, S305S had a milder effect on 4R expression, comparable to previously reported IVS10+16 mutations. Therefore, our limited period of neuronal differentiation (up to 8 weeks) may have precluded detection of more impactful effects on splicing that may occur as neurons further matured. We have carried out transcriptomic evaluation and targeted functional validation of phenotypes of interest in these cell lines; however, the mechanisms by which either mutant or 4R tau causes this disruption were not addressed in this study. We also focused our work on 2D neuronal and astrocytic monocultures. While this is valuable to understand cell-autonomous effects of *MAPT* mutations in neural cells, expansion of these studies to more complex 3D models and other neural cell types is required to fully understand the impact of *MAPT* S305 mutations in the brain. The generalizability of these data to sporadic tauopathy and other mutation models remains to be determined.

## RESOURCE AVAILABILITY

### Lead contact

Requests for further information and resources and reagents should be directed to the lead contact, Kathryn Bowles (kbowles@ed.ac.uk).

### Materials availability

Human iPSC lines described in this study can be requested from NCRAD (https://ncrad.iu.edu/) and the Tau Consortium via NSCI (http://neuralsci.org/tau).

### Data and code availability

- Bulk RNA-seq data and targeted *MAPT* ISOseq data generated as part of this study are available at the Gene Expression Omnibus (GEO: GSE260517), and the outputs of the analyses are available upon request. Other data generated from this study are available upon request.
- All code used in this study has been previously published, and the pipelines are described in the STAR Methods.
- Any additional information required to reanalyze the data reported in this paper is available from the lead contact upon request.

## STAR★METHODS

### EXPERIMENTAL MODEL AND SUBJECT DETAILS

#### Cell lines

WT/S305N (female) and WT/S305I (female) peripheral blood mononuclear cells were identified as part of the ALLFTD study (https://www.allftd.org/), and were requested and obtained from the National Centralized Repository for Alzheimer’s Disease (NCRAD; https://ncrad.iu.edu/). WT/S305S (male) lymphoblastoid cell lines (LCLs) were obtained from the Sydney Brain Bank (https://sbb.neura.edu.au/). PBMCs and LCLs were reprogrammed to iPSCs by the Icahn School of Medicine at Mount Sinai Stem Cell core facility, using Sendai virus mediated gene transfer of *OCT4*, *SOX2*, *KLF4* and c-*MYC*. Following reprogramming, karyotypic normality of three parent clones was confirmed by G-band karyotyping conducted by WiCell (www.wicell.org), and mutation status was confirmed by Sanger sequencing (see below for details). WT donor line F13505.1 (female) was obtained from the Knight Alzheimer’s Disease Research Center at Washington University. Expression of pluripotency markers was assessed by immunofluorescence (see below for details). The iPSCs were maintained in StemFlex media (ThermoFisher Scientific) in feeder-free culture in wells coated with Matrigel (Corning). Cells were cultured at 37°C and 5% CO_2._ Passaging of cultures was carried out using ReLeSR (StemCell Technologies).

### METHOD DETAILS

#### CRISPR/Cas9 genome editing

Human iPSCs were edited using the pSpCas9(BB)-2A-GFP (PX458) construct backbone (Addgene #48138) described in Ran *et al* 2013^82^. For WT correction of S305 mutation lines, specific guide RNAs (gRNAs) were designed against the mutant allele using the Zhang lab design tool (crispr.mit.edu), and guides with the highest quality scores and more than 3bp mismatches with other genomic loci were selected. For the generation of homozygous mutants and introduction of S305 mutations onto a WT control background, guides were designed against the WT allele with the same method. Guides were cloned into the CRISPR/Cas9 vector backbone using the Zhang lab protocol (addgene.org/crispr/zhang) and correct insertion was confirmed by Sanger sequencing using primers for the human U6 promoter. Validation of correct targeting and cutting activity was validated in HEK293T cells using the Surveyor nuclease assay (IDT) before use in iPSCs. Repair templates (single stranded oligodeoxynucleotides (ssODN)) were designed to span 70 nucleotides either side of the Cas9 cleavage site. ssODNs were designed with a synonymous protospacer adjacent motif (PAM) site modification (CCC to ACC) to reduce the chance of multiple cutting events (V300V; GUC > GUA, Figures S6A and S6B. This mutation was confirmed by RNA structure prediction by Drs. Chen and Disney to have no effect on exon 10-Intron 10 hairpin free energy, and therefore was not predicted to alter exon 10 splicing (Figure S6C). This was confirmed by introduction of the mutation into a *MAPT* exon 10 minigene^87^ by site directed mutagenesis and expression in SH-SY5Y cells (Figure S6D). S305S, S305N and S305I mutations all substantially increased exon 10 inclusion in this system (Figure S6D). The PAM site edit was not incorporated in all clones, therefore summary of PAM edit status is included in Table 1. All guide RNA sequences are provided in Table S1.

For editing, iPSCs were maintained in DMEM/F12 with 10% FBS and 10μM Y27632, without antibiotics, overnight. Cells were then dissociated into a single cell suspension in Accutase (Innnovative Cell Tech), and 2×10^6^ cells were electroporated with the Neon transfection system (ThermoFisher Scientific) in 100μl tips with 4μg gRNA-Cas9 vector and 40μM ssODN. Cells were electroporated at 1400V for 20ms, with 2 pulses. Electroporated cells were seeded into a 10cm dish in DMEM/F12, 10% FBS and 10μM Y27632 for recovery. After 24 hours, media was replaced daily with StemFlex (ThermoFisher Scientific) containing gradually reducing concentrations of Y27632. When recovered, 288 individual clones (3 × 96w plates) were selected using Collagenase (ThermoFisher Scientific) dissociation and manual selection under a microscope. Clones were seeded into 96-well plates for recovery and expansion. Each plate was then split 1:2 using ReLeSR (StemCell Technologies), with half of the cells used for DNA extraction for Sanger sequencing, and the other half cryopreserved until mutation status could be confirmed.

#### Clone validation

DNA was extracted from 96-well plates of cells using QuickExtract (Epicentre) by incubation at 65°C for 6 minutes, followed by 2 minutes at 98°C. The region spanning the targeted mutation was amplified using Phusion mastermix (Cell Signaling Technology) with the following primers: Forward 5’- GACTCAACCTCCCGTCACTC –3’, Reverse 5’- GGGACACCCCCTCCTAGAAT –3’. PCR products were confirmed by Tapestation (Agilent), and cleaned up with ExoSAP-IT (ThermoFisher Scientific). Forward and reverse sequencing reactions were carried out using 20ng of PCR product and BigDye Terminator v3.1, with the same primers used for PCR amplification. Before sequencing, products were cleaned up using the BigDye Xterminator purification kit (ThermoFisher Scientific) and were assessed using the SeqStudio Genetic Analyzer (ThermoFisher Scientific). Sequences were visualized using Chromas (https://technelysium.com.au/wp/chromas/).

Potential off-target edits were identified by the Zhang lab design tool (crispr.mit.edu), and the top two targets with the lowest degree of nucleotide mismatch and highest likelihood of editing were selected for validation; intron 5 of *SSH2* (Hg37,Chr17: 28016842–28016864) and exon 18 of *UNC45A* (Hg37,Chr15: 91492935–91492957) (Figures S6E and S6F). Primers for amplification and sequencing used were forward 5’-TCCCCTGCCTCTTTATTCCT-3’ reverse 5’-CCATGATGCCCTGCTAATTT-3’ for SSH2 and forward 5’-GTTCCATATCTAACACCCAGCTC-3’, reverse 5’-CCTGCCAGAGTCCCAGA-3’. All lines and clones were confirmed free of off targets (Table 1). Expression of pluripotency markers was assessed by immunofluorescence (see below for details).

To confirm relatedness of resulting clones from each parent line, DNA was extracted from the final clones using the Qiagen DNEasy blood & Tissue kit, and submitted for genotyping at the Center for Applied Genomics (CAG) on the Gene Set Array (GSA) chip. Genotype data were processed in PLINK^88,89^. Data was filtered to choose a single probe for each marker, and were filtered by variant to have Hardy-Weinberg equilibrium of ≥ 1×10^30^, no more than 5% missingness overall and by sample to have no more than 50% missingness per 10kb imputation chunk. Data was imputed using Topmed r2^90^ and filtered to include markers with at least an R2 of 0.3, missingness of no more than 2%, and minor allele frequency of at least 0.02. Identity by descent was determined using King^91^ (Figure S6G).

#### iPSC culture and differentiation

iPSCs were maintained on Matrigel (Corning)-coated plates in StemFlex (ThermoFisher Scientific) supplemented with 1% penicillin/streptomycin (ThermoFisher Scientific), and were passaged with ReLeSR (StemCell Technologies). iPSCs were differentiated to neural progenitor cells (NPCs) and were MACS sorted to deplete neural crest cells^92^. Briefly, neural crest cells were depleted by labelling with CD271 antibody-coated magnetic beads (Miltenyi), followed by gravity flow through depletion columns (Miltenyi) placed on a QuadroMacs magnet (Miltenyi). Neural crest cells were retained in the column, while NPCs were collected in the flow-through. The flow-through was then labelled with CD133 antibody-coated magnetic beads (Miltenyi), and passed through selection columns (Miltenyi). CD133+ cells were retained in the column, and collected by elution in order to further enrich for NPCs For neuronal differentiation, NPCs were incubated with BrainPhys (StemCell Technologies) supplemented with 1xB27 without vitamin A, 1xN2 (both ThermoFisher Scientific), 20ng/ml BDNF, 20ng/ml GDNF (both R&D), 200μM ascorbic acid, 250μg/ml cyclic AMP (both Sigma Aldrich) and 1% penicillin/streptomycin. Neurons were maintained for up to 8 weeks, with media changes every other day. For astrocyte differentiation, NPCs were seeded sparsely into Matrigel-coated plates and treated with Astrocyte media (ScienCell)^93^. Media was replenished every 2–3 days, and the resulting astrocytes were collected or used for experiments at 30 days of differentiation.

#### Immunofluorescence

Cells were fixed with 10% Formalin (Sigma Aldrich) for 15 minutes at room temperature, then washed three times with PBS. They were then incubated with 0.1% Triton X-100 in PBS for 30 minutes with gentle rocking, followed by three additional PBS washes, and incubation with 1% bovine serum albumin (BSA) in PBS for a minimum of 30 minutes at room temperature. Cells were then incubated with primary antibodies prepared in 1% BSA in PBS overnight at 4°C, followed by three PBS washes and incubation with the appropriate secondary antibodies at a 1:100 dilution in 1% BSA in PBS for a minimum of 2 hours at room temperature. Secondary antibodies were then removed, and cells were incubated for 10 minutes at room temperature with 300nM DAPI prepared in PBS. Three PBS washes were repeated, and cells were stored in PBS at 4°C until ready for imaging. For pluripotency confirmation, the following antibodies were used: TRA-1–81 (Cell Signaling Technologies 4745, 1:1000), SSEA4 (Cell Signaling Technologies 4755, 1:1000), TRA-1–60 (Cell Signaling Technologies 4746, 1:1000), Nanog (Cell Signaling Technologies D73G4, 1:400), SOX2 (Cell Signaling Technologies 3579S, 1:400) and OCT4A (Cell Signaling Technologies 2840S, 1:400). For neuronal characterization, the following antibodies were used: Da9 (total tau, kind gift from Dr. Peter Davies, 1:500), RD4 (4R tau, Millipore, 1:250), Map2 (Abcam, 1:1000), β3-tubulin (Sigma Aldrich, 1:200) and NeuN (Abcam, 1:1000). Secondary antibodies were AlexaFluors Goat-anti-mouse 488 and Donkey-anti-Rabbit 568, all used at 1:100 and purchased from ThermoFisher Scientific. Cells were imaged on an inverted Zeiss Observer widefield fluorescent microscope with a 20x objective.

#### SDS-PAGE gel electrophoresis and western blot

For whole protein lysate analysis, neurons were suspended in Cell Lysis Buffer (Cell Signaling Technology) supplemented with 10μM PMSF and 1x protease/phosphatase cocktail (ThermoFisher Scientific) and sonicated on ice, followed by centrifugation at 13,000 × g for 10 minutes at 4°C to pellet debris. Astrocytes were lysed with M-PER (ThermoFisher Scientific) supplemented with Protease/Phosphatase inhibitor (ThermoFisher Scientific). Protein concentration was determined by BCA assay (ThermoFisher Scientific).

For synaptosome and cytosolic fractions, cells were lysed in Syn-Per Synaptic Protein Extraction reagent (ThermoFisher Scientific) and centrifuged first at 1,200 × g for 10 minutes at 4°C to remove debris, then a further 15,000×g for 20 minutes at 4°C to pellet synaptic proteins. The pellet was resuspended in PBS and retained as the synaptosome fraction, while the remaining supernatant was the cytosolic fraction. Protein concentrations were determined by BCA assay.

10–20μg of protein per sample was incubated with 1x Reducing Agent and 1x LDS sample buffer (both ThermoFisher Scientific) at 70°C for 10 minutes, before being immediately loaded onto BOLT 4–16% Bis-Tris gels in 1x MES buffer (ThermoFisher Scientific). Electrophoresis was carried out for 25 minutes at 200V before blotting onto nitrocellulose membranes using the iBlot system (ThermoFisher Scientific). Membranes were blocked for a minimum of 30 minutes at room temperature in 5% milk in PBS-T or 5% BSA. Primary antibodies were prepared at the following dilutions in 5% milk in PBS-T or 5% BSA : Da9 (total tau) 1:500, PHF1 (p-tau_S396_) 1:500 (both kind gifts from Dr. Peter Davies), Synapsin1 1:1000 (Synaptic Systems 106011), Homer1 1:1000 (Synaptic Systems 160003), GAPDH 1:10,000 (Abcam ab181602), 1:1000 βIII-tubulin (Biolegend 801213), 1:10.000 MAP2 (Abcam ab32454), 1:1000 Tau12 (Millipore MAB2241), and 1:1000 GFAP (Aves Lab GFAP857977) were incubated with the membrane overnight at 4°C with rocking. Membranes were washed 3x with PBS-T. Membranes were then incubated with either HRP Goat Anti-Rabbit or HRP Horse Anti-Mouse secondary antibodies (Vector laboratories) at a dilution of 1:20,000 in 5% milk in PBS-T for one hour at room temperature with gentle rocking. Following three additional washes, membranes were then incubated with WesternBright ECL HRP substrate (Advansta) or SuperSignal^™^ West Pico (Thermofisher) for 3 minutes before imaging on a UVP ChemiDoc or iBright. For re-staining, blots were incubated in Restore PLUS stripping buffer (ThermoFisher Scientific) for 15 minutes at room temperature, followed by one wash in PBS and re-blocking.

#### qRTPCR

Cell pellets were collected in cold PBS by scraping, and RNA was extracted using the Qiagen RNeasy mini RNA extraction kit, then reverse transcribed using the high capacity RNA-to-cDNA kit (ThermoFisher Scientific). For qRTPCR of 3R and 4R *MAPT* transcripts, amplification was carried out using SybrGreen mastermix (ThermoFisher Scientific) with the following primers: *MAPT* 4R Forward 5’-CGGGAAGGTGCAGATAATTAA-3’, Reverse 5’-GCCACCTCCTGGTTTATGATG-3’; *MAPT* 3R Forward 5’-AGGCGGGAAGGTGCAAATA-3’, Reverse 5’-GCCACCTCCTGGTTTATGATG-3’.

#### RNA-seq

RNA was isolated as described above, and library preparation with poly-A selection followed by sequencing with 150 base pair paired-end reads was carried out at Genewiz. Sequenced reads were trimmed for Illumina TruSeq adapters, and aligned to the human hg38 genome using the STAR aligner^83^ for quantification of gene-level read counts. Genes were filtered by expression abundance with the edgeR package v3.40.0, with default counts of at least 10 in each category, 15 overall, and expression in 70% of samples in the smallest group. Library sizes were estimated by the trimmed mean of M-values normalization (TMM) method^94^ using the R edgR package v3.40.0. Differential gene expression was predicted by linear mixed model analysis using the variancePartition package v1.28.0^95^ in order to account for sample correlation in isogenic lines. False discovery rate (FDR) of the differential expression test was estimated using the Benjamini-Hochberg (BH) method^96^. Gene set enrichment analysis (GSEA) was performed using the Molecular Signatures Database (MSigDB) version 7.1 Gene Ontology (GO) annotations of canonical pathways, biological processes, cellular compartments and molecular function, as well as all of the C2 curated genesets, with 10,000 permutations using GOtest 1.0.8 (https://github.com/mw201608/GOtest/tree/a54f23aa7cafc8a74489a3c8989fddf747c92987). Exon 10 PSI values were calculated from aligned BAM files using Mixture of Isoforms (MISO) software^84^ with default parameters.

#### *MAPT* targeted ISOseq

RNA was submitted to the Icahn School of Medicine at Mount Sinai Genomics CoRE for single molecule real time (SMRT) isoform sequencing (ISOseq) on the PacBio RS II platform using the following primers: Forward 5’-ATG GAA GAT CAC GCT GGG AC-3’, Reverse 5’-GAG GCA GAC ACC TCG TCA G-3’. Raw sequencing reads were passed through the ISOseq3 pipeline to detect full-length transcripts expressed in each sample. Beginning with raw subreads, single consensus sequences were generated for each *MAPT* amplicon with a SMRT adapter on both ends of the molecule. SMRT adapter sequences were then removed and MAPT-specific primer sequences were identified to orient the isoforms. Isoforms were subsequently trimmed of poly(A) tails and concatemers were identified and removed. Isoform consensus sequences were then predicted using a hierarchical alignment and iterative cluster merging algorithm to align incomplete reads to longer sequences. Finally, clustered isoform sequences were polished using the arrow model and binned into groups of isoforms with predicted accuracy of either ≥ 0.99 (high quality) or < 0.99 (low quality). The resulting isoforms were aligned to hg38 using the GMAP aligner.^85^

#### MEA

NPCs were seeded at a density of 25,000 cells per well in 48-well CytoView plates (Axion Biosystems) and differentiated into neurons as described above. Every week between 2- and 8- weeks of differentiation, spontaneous activity was measured with a 10 minute recording on the Axion Maestro using default neural real time spontaneous activity settings, and data was processed using the Axion AxiS Software with default Neural activity settings.

#### Seahorse assays

NPCs were seeded at a density of 50,000 cells per well into Seahorse XF96 96-well plates (Agilent), and were differentiated into neurons as described above. Mitochondrial bioenergetics were assessed at 6 weeks of differentiation using the Mitochondrial Stress Test measured on a Seahorse XFe96 Analyzer (both Agilent). Stress tests were carried out according to manufacturer’s instructions and cycling parameters. Briefly, neuronal media was changed to XF DMEM (Agilent) containing 1mM pyruvate, 2mM glutamine and 10mM glucose. Cells were equilibrated to the new media for 1 hour at 37°C in the absence of CO_2_ prior to starting the assay. The plate underwent 3 mix/measurement cycles to establish baseline OCR, followed by treatment with 1.5μM oligomycin followed by an additional 3 cycles. Cells were then exposed to 0.5μM FCCP and measured over 3 cycles, then were finally treated with 0.5μM Rotenone/Antimycin A and OCR was measured over a final 3 cycles. After assay completion, culture media was removed and replaced with Methylene Blue and incubated for 1 hour at room temperature to fix and stain cells. Methylene Blue was removed and the plate was thoroughly washed with distilled water, before cells were lysed with 4% acetic acid in 40% methanol. The resulting de-stain was transferred to a new plate and absorbance at 595nm was measured using a Varioskan microplate reader (ThermoFisher). The resulting values were applied as a scaling factor in Seahorse Wave analyzer software (V2.6.3.5) to correct OCR measurements for cell density. All OCR calculations were generated by the Seahorse Wave software.

#### Tau uptake assays

Monomeric recombinant tau of each of the 6 major isoforms was purchased from rPeptide, and was labelled using the pHrodo Red Microscale Protein Labeling kit (ThermoFisher Scientific). 100μg protein (1mg/ml) was incubated with 100μM sodium bicarbonate. 3μl of 2mM pHrodo iFL STP ester reactive dye was added to the sample, and incubated at room temperature for 15 minutes. Excess dye was removed from the sample by centrifugation for 5 minutes at 1,000 × g through a spin column containing gel resin

For the tau uptake assay, iPSC-astrocytes were seeded into 96-well plates at a density of 50,000 cells per well. After 24 hours, cells were then incubated with astrocyte media containing 1.5μg of the relevant pHrodo-labeled tau isoform or the equivalent volume of PBS. Treated plates were immediately placed into the IncuCyte live cell imaging system (EssenBio), and three fields of view per well were imaged every hour for 48 hours with a 20x objective. Image analysis was carried out in IncuCyte software for automated measurement of red integrated intensity and cell confluence.

#### ELISAs

Following exogenous tau treatment and imaging, conditioned media was collected from iPSC-astrocytes for assessment of CXCL5 secretion by sandwich ELISA (Abcam ab212163) according to manufacturer’s instructions. Absorbance was measured on a Varioskan microplate reader (ThermoFisher Scientific) and normalized to cell confluence as measured by Incucyte imaging at the time of media collection. CXCL5 concentration was determined by comparison to a positive control standard curve.

### QUANTIFICATION AND STATISTICAL ANALYSIS

RNA-seq and immunofluorescence data were analyzed as described above. Western blot protein bands were quantified by densitometry analysis in ImageJ, and normalized to GAPDH for each sample. For ISOseq data, the expression of each isoform was calculated as a proportion of all detected isoforms in that sample, and average expression for each genotype was calculated. qRTPCR gene expression was analyzed using the ΔΔCt method, and expression was normalized to β-actin as endogenous controls, then a ratio between 4R and 3R expression was calculated for each sample. Comparisons across time and genotypes were carried out using the appropriate two-way ANOVA and post-hoc testing, and comparisons between genotypes at single time points were carried out using one-way ANOVA and post-hoc comparisons for multiple test correction. Incucyte time course data was analyzed using simple linear models. Methods to determine whether data met assumptions of statistical approaches were not applied. All analyses were carried out in either GraphPad Prism 9, or R v4.2.2. For cell culture experiments, tests were either conducted on all available clones, or on one clone per genotype per parent line with three independent rounds of differentiation, averaged from multiple technical replicates. Specific statistical tests and *n* for individual assays are described in the relevant figure legends.

## Supplementary Material

1

2

3

4

5

6

7

SUPPLEMENTAL INFORMATION

Supplemental information can be found online at https://doi.org/10.1016/j.celrep.2024.115013.

## Figures and Tables

**Table 1.** Description of isogenic sets of *MAPT* S305 mutation iPSC lines

Parent ID	Clone ID	Donor genotype	Isogenic genotype	Description	Initial cell type	Source repository	iPSC available from	Sex	PAM edit	Off-target analysis	Pluripotency confirmed

75.11	–	WT/S305I	–	reprogrammed clone	PBMC	NCRAD	NCRAD	XX	–	–	Y
75.11	IW1A12	WT/S305I	WT/WT	corrected control	–	–	NCRAD	XX	Het	Y	Y
75.11	IW1B5	WT/S305I	WT/WT	corrected control	–	–	NCRAD	XX	none	Y	Y
75.11	IW1F3	WT/S305I	WT/WT	corrected control	–	–	NCRAD	XX	none	Y	Y
75.11	IW1G8	WT/S305I	WT/WT	corrected control	–	–	NCRAD	XX	none	Y	Y
75.11	IH1A8	WT/S305I	WT/S305I	unedited clone	–	–	NCRAD	XX	none	Y	Y
75.11	IH1A5	WT/S305I	WT/S305I	unedited clone	–	–	NCRAD	XX	none	Y	Y
75.11	IH1A4	WT/S305I	S305I/S305I	homozygous edit	–	–	NCRAD	XX	Hom	Y	Y
300.12	–	WT/S305N	–	reprogrammed clone	PBMC	NCRAD	NCRAD	XX	–	–	Y
300.12	NWBB7	WT/S305N	WT/WT	corrected control	–	–	NCRAD	XX	Het	Y	Y
300.12	NW1B10	WT/S305N	WT/S305N	unedited clone	–	–	NCRAD	XX	none	Y	Y
300.12	NW1C7	WT/S305N	WT/S305N	unedited clone	–	–	NCRAD	XX	none	Y	Y
300.12	NH1E7	WT/S305N	WT/S305N	unedited clone	–	–	NCRAD	XX	Het	Y	Y
300.12	HBE10	WT/S305N	S305N/S305N	homozygous edit	–	–	NCRAD	XX	none	Y	Y
300.12	HCB8	WT/S305N	S305N/S305N	homozygous edit	–	–	NCRAD	XX	Hom	Y	Y
300.12	HAE11	WT/S305N	S305N/S305N	homozygous edit	–	–	NCRAD	XX	none	Y	Y
GP1.1	–	WT/S305S	–	reprogrammed clone	lymphoblast	SBB	NeuraCell	XY	–	–	Y
GP1.1	WAC11	WT/S305S	WT/WT	corrected control	–	–	NeuraCell	XY	Hom	Y	Y
GP1.1	SW1B4	WT/S305S	WT/S305S	unedited clone	–	–	NeuraCell	XY	none	Y	Y
GP1.1	SW1D8	WT/S305S	WT/S305S	unedited clone	–	–	NeuraCell	XY	none	Y	Y
GP1.1	SH1G8	WT/S305S	S305S/S305S	homozygous edit	–	–	NeuraCell	XY	Hom	Y	Y
F13505.1	–	WT/WT	–	parent line	fibroblast	WashU ADRC	NeuraCell	XX	–	–	Y
F13505.1	S2A3	WT/WT	WT/WT	unedited clone	–	–	NeuraCell	XX	none	Y	Y
F13505.1	NEA11	WT/WT	WT/WT	unedited clone	–	–	NeuraCell	XX	Hom	Y	Y
F13505.1	I1B10	WT/WT	WT/S305I	heterozygous edit	–	–	NeuraCell	XX	Het	Y	Y
F13505.1	NDG11	WT/WT	WT/S305N	heterozygous edit	–	–	NeuraCell	XX	Het	Y	Y

PBMC, peripheral blood mononuclear cell; WT, wild-type; Het, heterozygous; Hom, homozygous; PAM, protospacer-adjacent motif; Y, yes.

**KEY RESOURCES TABLE:** 

REAGENT or RESOURCE	SOURCE	IDENTIFIER

Antibodies		

DAPI	Thermo Fisher Scientific	D1306
TRA-1-81	Cell Signaling Technologies	Cat#4745; RRID:AB_2119060
SSEA4	Cell Signaling Technologies	Cat#4755; RRID:AB_1264259
TRA-1-60	Cell Signaling Technologies	Cat#4746; RRID:AB_2119059
Nanog	Cell Signaling Technologies	Cat#4903; RRID:AB_10559205
SOX2	Cell Signaling Technologies	Cat#3579; RRID:AB_2195767
OCT4A	Cell Signaling Technologies	Cat#2840; RRID:AB_2167691
AlexaFluor Goat-anti-mouse 488	ThermoFisher Scientific	A-21141
AlexaFluor Donkey-anti-Rabbit 568	Life Technologies	A10042
Da9	Dr. Peter Davies	N/A
PHF1	Dr. Peter Davies	N/A
Synapsin1	Synaptic Systems	Cat#106011; RRID:AB_2619772
Homer1	Synaptic Systems	Cat#160003; RRID:AB_887730
GAPDH	Abcam	Cat#ab181602; RRID:AB_2630358
RD4	Millipore	Cat#05-804; RRID:AB_11211556
MAP2	Abcam	Cat#ab32454; RRID:AB_776174
NeuN	Abcam	Cat#ab104224; RRID:AB_10711040
βIII-tubulin	Sigma Aldrich	Cat#T8660; RRID:AB_477590
Tau12	Millipore	Cat#MAB2241; RRID:AB_1977340
GFAP	Aves Lab	Cat# GFAP857977: RRID:AB_2313547
βIII-tubulin	BioLegend	Cat#801213: RRID:AB_2728521
MAP2	Abcam	Cat# ab5392: RRID:AB_2138153
HRP Goat Anti-Rabbit	Vector laboratories	PI-1000
HRP Horse Anti-Mouse	Vector laboratories	PI-2000

Bacterial and virus strains		

pSpCas9(BB)-2A-GFP (PX458)	Ran *et al* 2013^82^	Addgene #48138

Chemicals, peptides, and recombinant proteins		

DMEM/F-12 with soduim pyruvate GlutaMAX^™^	ThermoFisher Scientific	10565018
FBS - heat-inactivated - mESC tested	Stem Cell Core	F4135-500ml
Y27632	BD Biosciences	562822
Accutase	Life Technologies	a11105-01
StemFlex	ThermoFisher Scientific	A3349401
Collagenase IV	ThermoFisher Scientific	17104019
ReLeSR	StemCell Technologies	05873
QuickExtract	Lucigen	QE09050
Phusion mastermix	Fisher Scientific	M0531L
ExoSAP-IT	ThermoFisher Scientific	75001.4X.1.ML
BigDye Terminator v3.1	Applied Biosystems	4337455
BigDye Xterminator	ThermoFisher Scientific	4376487
Corning^®^ Matrigel^®^ Growth Factor Reduced (GFR) Basement Membrane Matrix, *LDEV-Free	Corning	CB-40230
penicillin/streptomycin	ThermoFisher Scientific	15140122
BrainPhys	StemCell Technologies	05790
B27 without vitamin A	ThermoFisher Scientific	12-587-010
N2	ThermoFisher Scientific	17502048
BDNF	R&D	248-BD-025
GDNF	R&D	212-GD-050
ascorbic acid	Sigma Aldrich	A0278-100G
cyclic AMP	Sigma Aldrich	D0627-1G
Astrocyte media	ScienCell	1801
Formalin	Sigma Aldrich	HT5011-1CS
Triton X-100	Sigma Aldrich	X100-100ML
bovine serum albumin	Sigma Aldrich	A9647-50G
Cell Lysis Buffer	Cell Signaling Technology	9803S
M-PER	ThermoFisher Scientific	78501
PMSF	Sigma Aldrich	93482-50ML-F
protease/phosphatase cocktail	ThermoFisher Scientific	5872S
Syn-Per Synaptic Protein Extraction reagent	ThermoFisher Scientific	87793
M-Per	ThermoFisher Scientific	78501
Invitrogen Novex 10X Bolt Sample Reducing Agent	ThermoFisher Scientific	B0009
LDS sample buffer	ThermoFisher Scientific	B0007
MES buffer	ThermoFisher Scientific	41320040-2
WesternBright ECL HRP substrate	Advansta	K-12045-D20
SuperSignal^™^ West Pico PLUS Chemiluminescent Substrate	ThermoFisher Scientific	34580
Restore PLUS stripping buffer	Scientific	PI46430
0N3R tau	rPeptide	T-1006-2
0N4R tau	rPeptide	T-1005-2
1N3R tau	rPeptide	T-1004-1
1N4R tau	rPeptide	T-1003-2
2N3R tau	rPeptide	T-1002-2
2N4R tau	rPeptide	T-1001-1

Critical commercial assays		

pHrodo^™^ Red Microscale Labeling Kit	ThermoFisher Scientific	P35363

Deposited data		

Bulk RNA-seq data from *MAPT* S305 mutation neurons and astrocytes	This study	GEO: GSE260517 https://www.ncbi.nlm.nih.gov/geo/query/acc.cgi?acc=GSE260517
Targeted MAPT ISO-seq data from MAPT S305 mutation neurons and astrocytes	This study	GEO: GSE260517 https://www.ncbi.nlm.nih.gov/geo/query/acc.cgi?acc=GSE260517

Experimental models: Cell lines		

75.11	NCRAD	https://ncrad.iu.edu
75.11-IW1A12	NCRAD	https://ncrad.iu.edu/
75.11-IW1B5	NCRAD	https://ncrad.iu.edu
75.11-IW1F3	NCRAD	https://ncrad.iu.edu
75.11-IW1G8	NCRAD	https://ncrad.iu.edu
75.11-IH1A8	NCRAD	https://ncrad.iu.edu
75.11-IH1A5	NCRAD	https://ncrad.iu.edu
75.11-IH1C10	NCRAD	https://ncrad.iu.edu
75.11-IH1B9	NCRAD	https://ncrad.iu.edu
75.11-IH1A4	NCRAD	https://ncrad.iu.edu
300.12	NCRAD	https://ncrad.iu.edu
300.12-NWBB7	NCRAD	https://ncrad.iu.edu
300.12-NW1B10	NCRAD	https://ncrad.iu.edu
300.12-NW1C7	NCRAD	https://ncrad.iu.edu
300.12-NH1E7	NCRAD	https://ncrad.iu.edu
300.12-HBE10	NCRAD	https://ncrad.iu.edu
300.12-HCB8	NCRAD	https://ncrad.iu.edu
300.12-HAE11	NCRAD	https://ncrad.iu.edu
GP1.1	NeuraCell	https://www.neuralsci.org/tau/human-ipsc-lines
GP1.1-WAC11	NeuraCell	https://www.neuralsci.org/tau/human-ipsc-lines
GP1.1-SW1B4	NeuraCell	https://www.neuralsci.org/tau/human-ipsc-lines
GP1.1-SW1D8	NeuraCell	https://www.neuralsci.org/tau/human-ipsc-lines
GP1.1-SH1F6	NeuraCell	https://www.neuralsci.org/tau/human-ipsc-lines
GP1.1-SH1G8	NeuraCell	https://www.neuralsci.org/tau/human-ipsc-lines
F13505.1	NeuraCell	https://www.neuralsci.org/tau/human-ipsc-lines
F13505.1-S2A3	NeuraCell	https://www.neuralsci.org/tau/human-ipsc-lines
F13505.1-I1E12	NeuraCell	https://www.neuralsci.org/tau/human-ipsc-lines
F13505.1-NEA11	NeuraCell	https://www.neuralsci.org/tau/human-ipsc-lines
F13505.1-I1B10	NeuraCell	https://www.neuralsci.org/tau/human-ipsc-lines
F13505.1-NDG11	NeuraCell	https://www.neuralsci.org/tau/human-ipsc-lines
F13505.1-S3H5	NeuraCell	https://www.neuralsci.org/tau/human-ipsc-lines

Oligonucleotides		

Primers for CRISPR/Cas9 gRNA targeting of *MAPT* S305, see Table S1	This paper	N/A
U6 promoter primer for Sanger sequencing	Addgene	https://www.addgene.org/mol-bioreference/sequencing-primers/
Additional sequencing & qRTPCR primer sequences	This paper	Table S6

Software and algorithms		

CRISPR design tool	Zhang Lab, MIT	crispr.mit.edu (now unavailable) https://www.zlab.bio/resources
STAR aligner	Dobin *et al* 2013^83^	https://github.com/alexdobin/STAR
Ingenuity Pathway Analysis	Qiagen Inc	https://digitalinsights.qiagen.com/productsoverview/discovery-insights-portfolio/analysisand-visualization/qiagen-ipa/
Mixture of Isoforms (MISO)	Katz et al 2010^84^	https://miso.readthedocs.io/en/fastmiso/
GMAP aligner	Wu et al 2005^85^	https://github.com/juliangehring/GMAP-GSNAP/blob/master/README
Axion AxiS	Axion Biosystems	N/A
Seahorse Wave analyzer V2.6.3.5	Agilent	https://www.agilent.com/en/product/cell-analysis/real-time-cell-metabolicanalysis/xf-software/seahorse-wavedesktop-software-740897
Prism 9	GraphPad	https://www.graphpad.com/
R v4.2.2	R Foundation	https://www.r-project.org/
ImageJ	Schneider et al 2012^86^	https://imagej.net/ij/

## Highlights

- Characterization of 26 human iPSC clones with three different *MAPT* splicing mutations
- S305 mutations efficiently increase 4R tau expression in iPSC neurons and astrocytes
- Altered oxidative phosphorylation and spontaneous firing are observed in neurons
- Inflammation and rapidly internalized exogenous tau are observed in astrocytes
