# Supplementary material for: Development of MAPT S305 mutation human iPSC lines exhibiting elevated 4R tau expression and functional alterations in neurons and astrocytes
Source: Cell Rep. Author manuscript; Available in PMC 2026 Aug 17. (PMC13478741; doi:10.1016/j.celrep.2024.115013)
Supplement: Supplementary file 1 [file NIHMS2159651-supplement-1.pdf]

Cell Reports, Volume *43*

Supplemental information

Development of MAPT S305 mutation human iPSC lines exhibiting elevated 4R tau expression and functional alterations in neurons and astrocytes

Kathryn R. Bowles, Chiara Pedicone, Derian A. Pugh, Laura-Maria Oja, Filipa H. Sousa, Lois K. Keavey, Brian Fulton-Howard, Sarah A. Weitzman, Yiyuan Liu, Jonathan L. Chen, Matthew D. Disney, and Alison M. Goate

Development of *MAPT* S305 mutation models exhibiting elevated 4R tau expression, resulting in altered neuronal and astrocytic function

KR Bowles[1,2*•], C Pedicone[1,2], DA Pugh[1,2Ŧ], L Oja[1,2#], F Henderson-Sousa[3], LK Keavey[3], B Fulton-Howard[1,2], SA Weitzman[1,2], Y Liu[1,2], JL Chen[4+], MD Disney[4] & AM Goate[1,2]

Supplementary Figures

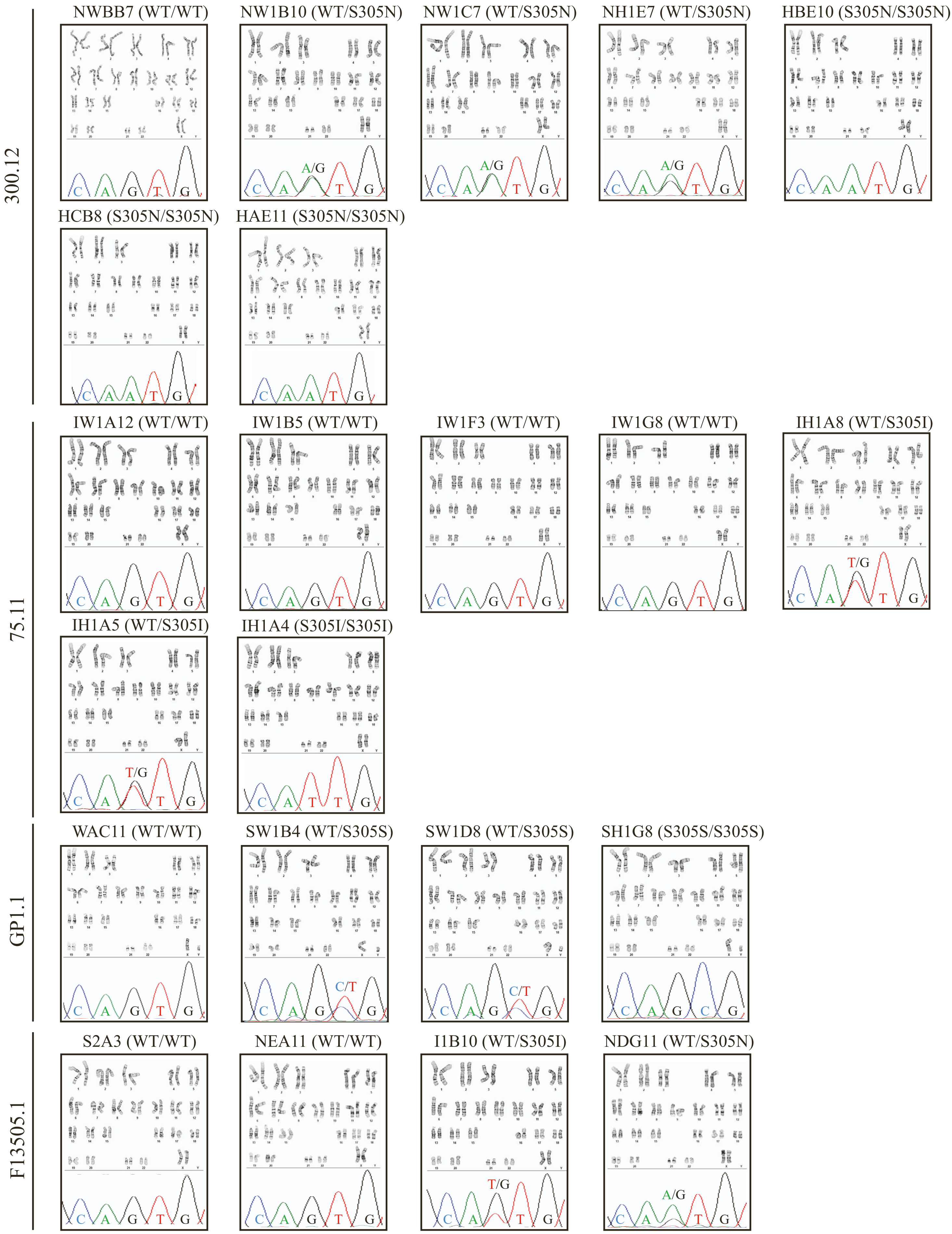
300.12
NWBB7 (WT/WT)
NW1B10 (WT/S305N)
NW1C7 (WT/S305N)
NH1E7 (WT/S305N)
HBE10 (S305N/S305N)
HCB8 (S305N/S305N)
HAE11 (S305N/S305N)
75.11
IW1A12 (WT/WT)
IW1B5 (WT/WT)
IW1F3 (WT/WT)
IW1G8 (WT/WT)
IH1A8 (WT/S305I)
IH1A5 (WT/S305I)
IH1A4 (S305I/S305I)
GP1.1
WAC11 (WT/WT)
SW1B4 (WT/S305S)
SW1D8 (WT/S305S)
SH1G8 (S305S/S305S)
F13505.1
S2A3 (WT/WT)
NEA11 (WT/WT)
I1B10 (WT/S305I)
NDG11 (WT/S305N)

Figure S1. Karyotyping and sequencing of CRISPR-edited clones (related to Figure 1)

G-band karyotyping and Sanger sequencing traces from all CRISPR-edited and non-edited clonal controls generated from the four parent iPSC lines.

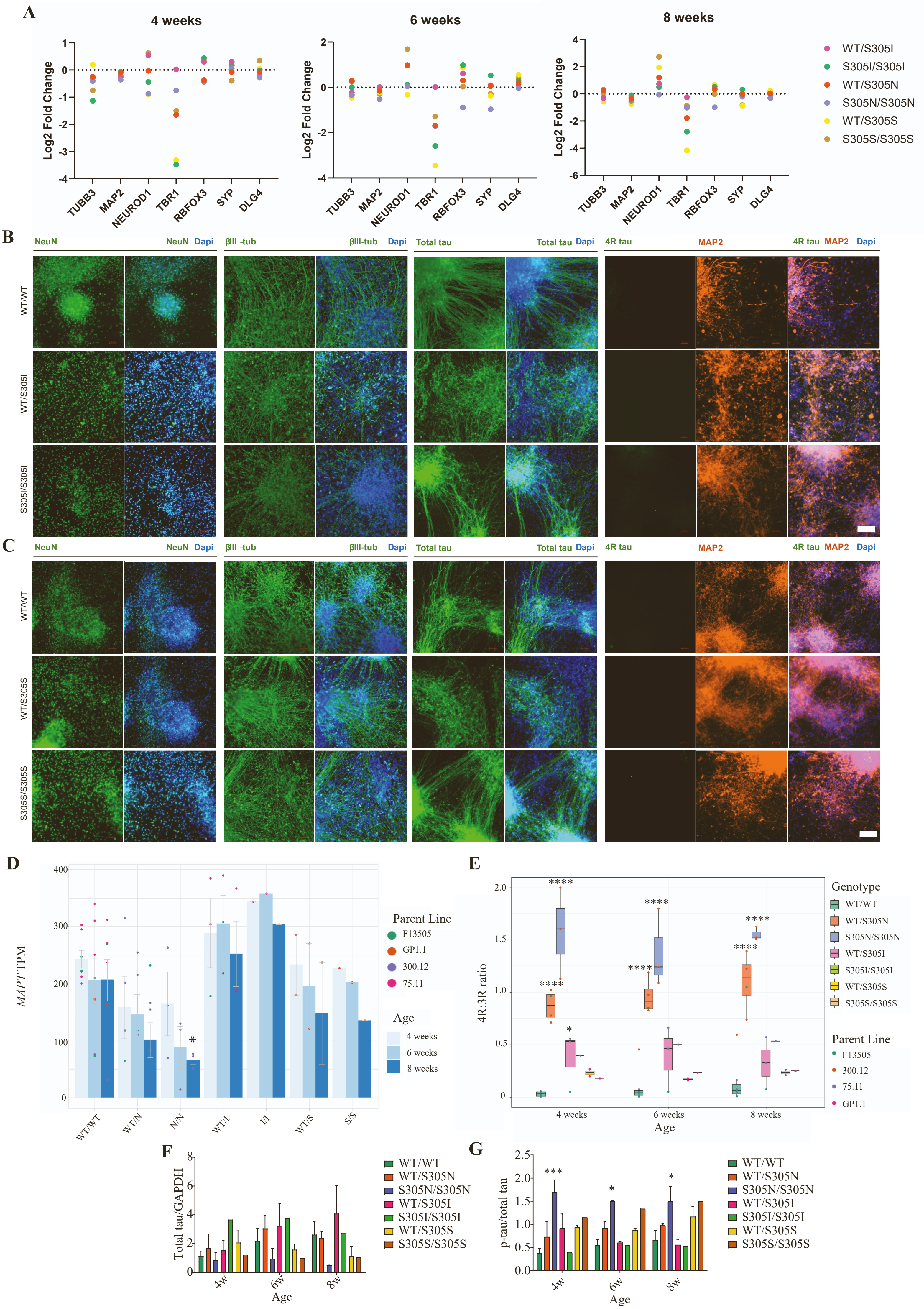

A
4 weeks
6 weeks
8 weeks
Log2 Fold Change
TUBB3
MAP2
NEUROD1
TBR1
RBFOX3
SYP
DLG4
WT/S305I
S305I/S305I
WT/S305N
S305N/S305N
WT/S305S
S305S/S305S
B
NeuN
NeuN Dapi
βIII -tub
βIII-tub Dapi
Total tau
Total tau Dapi
4R tau
MAP2
4R tau MAP2 Dapi
WT/WT
WT/S305I
S305I/S305I
C
WT/S305S
S305S/S305S
D
MAPT TPM
Parent Line
F13505
GP1.1
300.12
75.11
Age
4 weeks
6 weeks
8 weeks
WT/WT
WT/N
N/N
WT/I
I/I
WT/S
S/S
E
4R:3R ratio
Genotype
WT/WT
WT/S305N
S305N/S305N
WT/S305I
S305I/S305I
WT/S305S
S305S/S305S
Parent Line
F13505
300.12
75.11
GP1.1
Age
F
Total tau/GAPDH
4w
6w
8w
G
p-tau/total tau

Figure S2. ***MAPT*** **S305 mutations have both shared and divergent effects on Exon 10 inclusion and differentially expressed gene pathway enrichment (related to Figures 2 and 3).**

A. Log2 fold change expression of neuronal maturation markers in S305 mutation iPSC-neurons compared to isogenic controls at 4, 6 and 8 weeks of differentiation

B-C. Representative images of neuronal marker and tau staining in **B.** S305I and **C.** S305S mutation neurons and isogenic controls at 4 weeks of differentiation. Scale bar = 100µm

D. *MAPT* expression (transcripts per million, TPM) from RNA-seq data of 4, 6 and 8 week old iPSC-neurons per clone, separated by mutation. Error bars = SEM. N = 1-7. Two-way mixed model ANOVA, correcting for Parent Line, with Dunnett's post-hoc test correction.

E. 4R:3R ratio calculated from qRTPCR analysis of Exon 10+ and Exon 10- transcripts across all clones at each time point. Error bars = SEM. N = 1-7. Two-way ANOVA. *p < 0.05, **p < 0.01, ***p < 0.001, ****p < 0.0001.

F-G. Quantification of total tau and p-tau (S396), normalized to GAPDH across all mutations and time points, as represented in ***Figure 2D.*** Error bars = SEM. Two-way ANOVA with Bonferroni post-hoc testing compared to isogenic controls

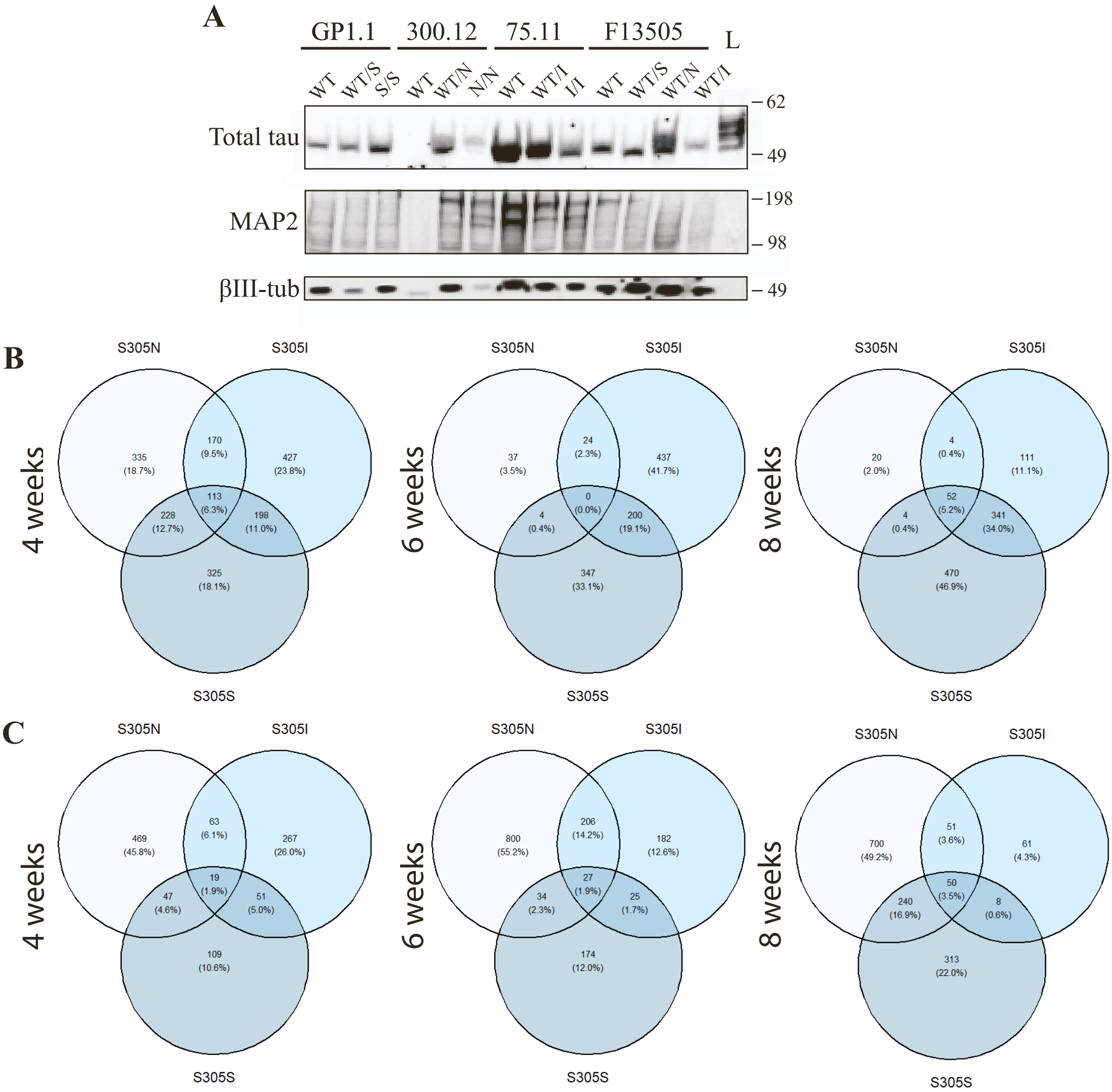
A
GP1.1
300.12
75.11
F13505
L
WT
WT/S
S/S
WT
WT/N
N/N
WT
WT/I
I/I
WT
WT/S
WT/N
WT/I
Total tau
MAP2
βIII-tub
62
49
198
98
49
B
C
S305N
S305I
S305S
4 weeks
6 weeks
8 weeks
335 (18.7%)
170 (9.5%)
427 (23.8%)
113 (6.3%)
228 (12.7%)
198 (11.0%)
325 (18.1%)
37 (3.5%)
24 (2.3%)
437 (41.7%)
0 (0.0%)
4 (0.4%)
200 (19.1%)
347 (33.1%)
20 (2.0%)
4 (0.4%)
111 (11.1%)
52 (5.2%)
4 (0.4%)
341 (34.0%)
470 (46.9%)
469 (45.8%)
63 (6.1%)
267 (26.0%)
19 (1.9%)
47 (4.6%)
51 (5.0%)
109 (10.6%)
800 (55.2%)
206 (14.2%)
182 (12.6%)
27 (1.9%)
34 (2.3%)
25 (1.7%)
174 (12.0%)
700 (49.2%)
51 (3.6%)
61 (4.3%)
50 (3.5%)
240 (16.9%)
8 (0.6%)
313 (22.0%)

Figure S3. ***MAPT*** **S305 mutations increase 4R tau and elicit numerous transcriptomic changes in neurons (related to Figures 2 and 3).**

A. Dephoshorylated protein lysate from S305 mutation neurons at 8 weeks of differentiation, probed for total tau and neuronal markers MAP2 and βIII-tubulin. L = tau ladder.

B-C. Overlap of the number of significantly enriched pathways from GSEA analysis between S305 ***B.*** heterozygous and ***C.*** homozygous mutations at each time point, compared to isogenic controls.

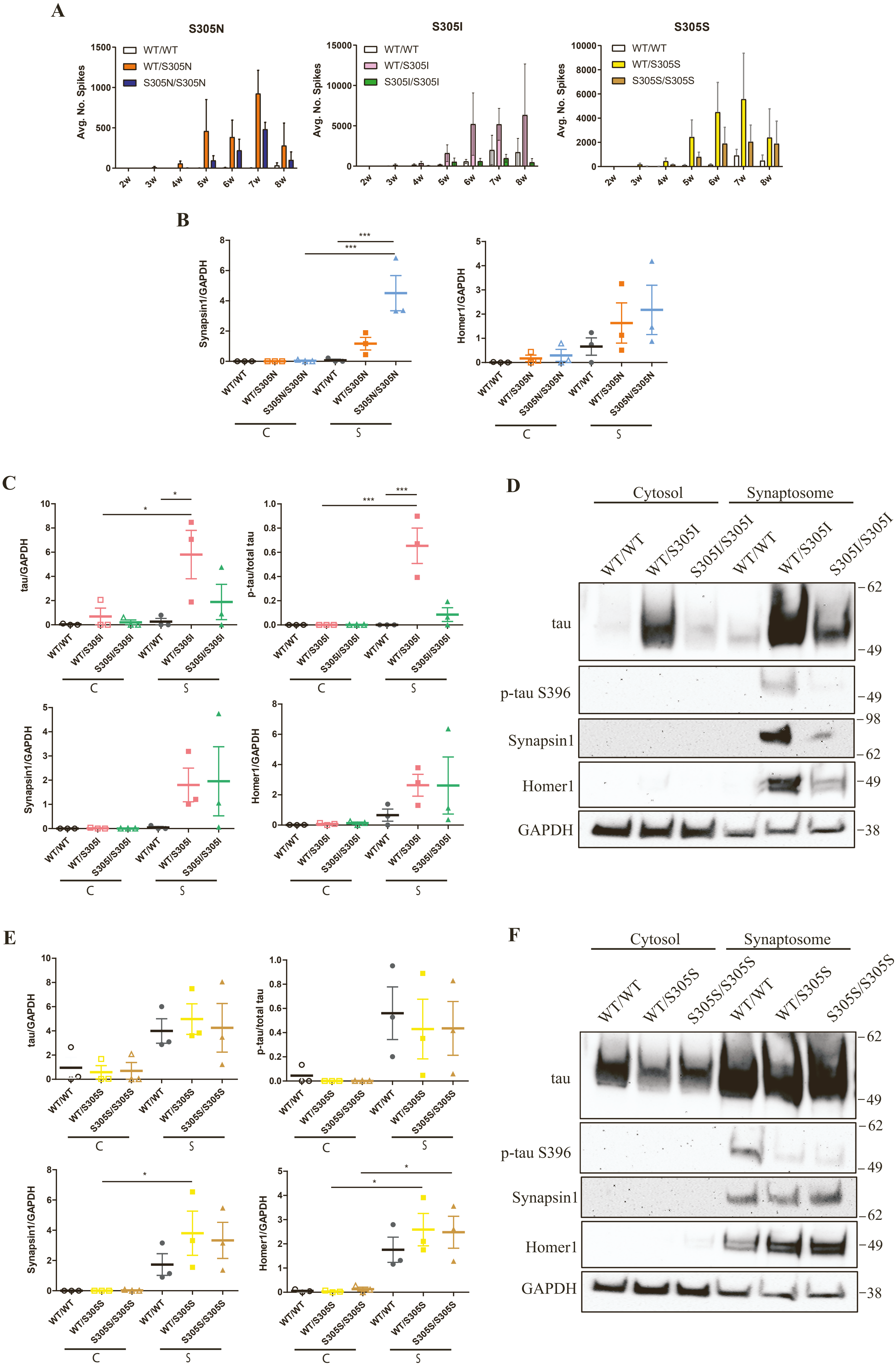

A
S305N
Avg. No. Spikes
WT/WT
WT/S305N
S305N/S305N
S305I
WT/S305I
S305I/S305I
S305S
WT/S305S
S305S/S305S
2w
3w
4w
5w
6w
7w
8w
B
Synapsin1/GAPDH
Homer1/GAPDH
C
S
C
tau/GAPDH
p-tau/total tau
D
Cytosol
Synaptosome
tau
p-tau S396
Synapsin1
Homer1
GAPDH
E
F

Figure S4. Neuronal synaptic maturity is accelerated in *MAPT* S305 mutation lines (related to Figure 3)

A. Average number of spikes derived from multi-electrode array analysis of isogenic sets of S305N, S305I and S305S iPSC-neurons every week from 3-8 weeks of age. Error bars = SEM. N = 3 independent differentiations, averaged from 3 technical replicates per genotype/clone. Two-way ANOVA.

B. Quantification of western blot for Synapsin1 and Homer1 in Figure 3F, normalized to GAPDH loading control in 6-week old S305N mutation cytosol (C) and synaptosome (S) fractions. Error bars = SEM. N = 3. One way ANOVA with Bonferroni post-hoc testing.

C. Quantification of western blot as represented in ***D,*** for total tau, p-tau (S396), Synapsin1 and Homer1, normalized to GAPDH loading control in 6-week old S305I mutation cytosol (C) and synaptosome (S) fractions compared to isogenic controls. Error bars = SEM. N = 3. One way ANOVA with Bonferroni post-hoc testing.

D. Representative western blot of cytosolic and synaptosome fractions derived from 6-week old S305I mutation neurons and isogenic controls, labelled for total tau and phospho-tau, as well as synaptic markers Synapsin1 and Homer1. GAPDH used as loading control.

E. Quantification of western blots as represented in ***F,*** for total tau, p-tau (S396), Synapsin1 and Homer 1, normalized to GAPDH loading control in 6-week old S305S mutation cytosol (C) and synaptosome (S) fractions compared to isogenic controls. Error bars = SEM. N = 3. One way ANOVA with Bonferroni post-hoc testing.

F. Representative western blot of cytosolic and synaptosome fractions derived from 6-week old S305S mutation neurons and isogenic controls, labelled for total tau and phospho-tau, as well as synaptic markers Synapsin1 and Homer1. GAPDH used as loading control.

$^{*}p < 0.05$, $^{**}p < 0.01$, $^{***}p < 0.001$

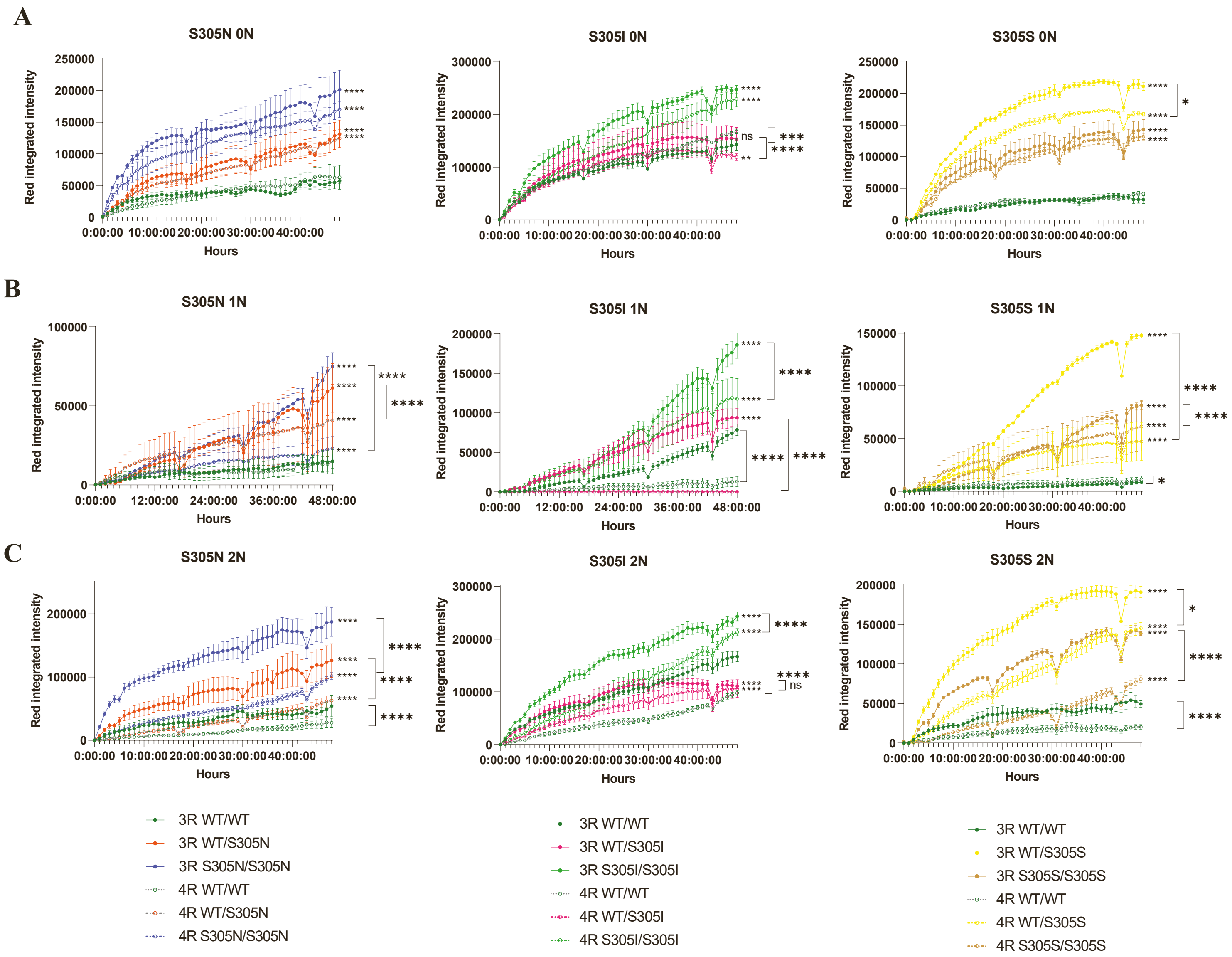

Figure S5. iPSC-astrocytes with *MAPT* S305 mutations have altered tau internalization (related to Figure 6).

A. Representative trace of integrated fluorescence intensity of pHrodo Red signal in S305 mutation astrocytes and isogenic controls treated with labelled 0N3R and 0N4R tau isoforms over 48 hours. Error bars = SEM. N = 4, each point is an average of 3 fields of view per well. Linear regression.

B. Representative trace of integrated fluorescence intensity of pHrodo Red signal in S305 mutation astrocytes and isogenic controls treated with labelled 1N3R and 1N4R tau isoforms over 48 hours. Error bars = SEM. N = 4, each point is an average of 3 fields of view per well. Linear regression.

C. Representative trace of integrated fluorescence intensity of pHrodo Red signal in S305 mutation astrocytes and isogenic controls treated with labelled 2N3R and 2N4R tau isoforms over 48 hours. Error bars = SEM. N = 4, each point is an average of 3 fields of view per well. Linear regression.

*p < 0.05, **p < 0.01, ***p < 0.001, ****p < 0.0001.

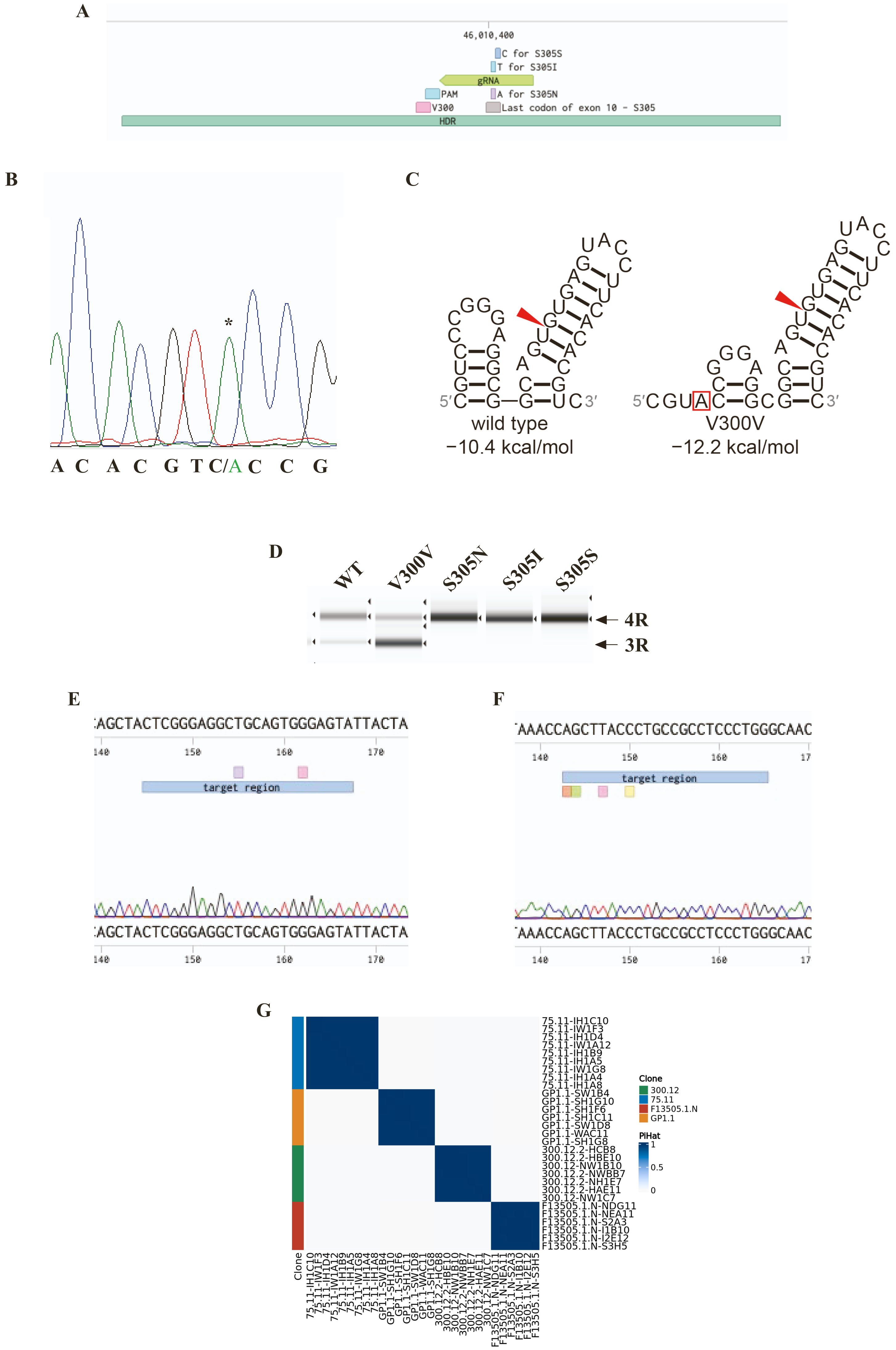

A
46,010,400
C for S305S
T for S305I
gRNA
PAM
A for S305N
V300
Last codon of exon 10 - S305
HDR
B
*
A C A C G T C/A C C G
C
wild type
−10.4 kcal/mol
V300V
−12.2 kcal/mol
D
WT
V300V
S305N
S305I
S305S
4R
3R
E
AGCTACTCGGGAGGCTGCAGTGGGAGTATTACTA
140 150 160 170
target region
F
AAACCAGCTTACCCTGCCGCCTCCCTGGGCAAC
140 150 160
target region
G
75.11-IH1C10
75.11-IW1F3
75.11-IH1D4
75.11-IW1A12
75.11-IH1B9
75.11-IH1A5
75.11-IW1G8
75.11-IH1A4
75.11-IH1A8
GP1.1-SW1B4
GP1.1-SH1G10
GP1.1-SH1F6
GP1.1-SH1C11
GP1.1-SW1D8
GP1.1-WAC11
GP1.1-SH1G8
300.12.2-HCB8
300.12.2-HBE10
300.12-NW1B10
300.12.2-NWBB7
300.12.2-NH1E7
300.12.2-HAE11
300.12-NW1C7
F13505.1.N-NDG11
F13505.1.N-NEA11
F13505.1.N-S2A3
F13505.1.N-I1B10
F13505.1.N-I2E12
F13505.1.N-S3H5
Clone
300.12
75.11
F13505.1.N
GP1.1
PiHat
1
0.5
0

Figure S6. Edited iPSC lines QC, related to Figure 1 and Methods)

A. Schematic of gRNA design

B. Sanger sequencing trace indicating V300V PAM site mutation (GUC > GUA). Modified base is highlighted with an asterisk.

C. Predicted free energies (kcal) of structures at the Exon 10 – Intron 10 hairpin loop in WT and PAM site mutation V300V sequences. The Exon-Intron junction is indicated with an arrow and the V300V modification is indicated with a red box. The structure of the WT sequence was reported by Lisowiec *et al*.[81] V300V mutant structure was predicted using RNAstructure.[91]

D. Representative RTPCR of *MAPT* Exon 10 minigenes edited with V300V or S305 mutations, expressed in SH-SY5Y cells. Top band is 4R tau, bottom band is 3R tau.

E. Representative sequencing trace for off target sequence on intron 5 of *SSH2*

F. Representative sequencing trace for off target sequence on exon 18 of *UNC45A*

G. Relatedness of edited iPSC clones
